# DRSegNet: A cutting-edge approach to Diabetic Retinopathy segmentation and classification using parameter-aware Nature-Inspired optimization

**DOI:** 10.1371/journal.pone.0312016

**Published:** 2024-12-05

**Authors:** Sundreen Asad Kamal, Youtian Du, Majdi Khalid, Majed Farrash, Sahraoui Dhelim

**Affiliations:** 1 School of Electronics and Information Technology, Xi’an Jiaotong University, Xian, China; 2 Department of Computer Science and Artificial Intelligence, College of Computing, Umm Al-Qura University, Makkah, Saudi Arabia; 3 Dublin City University, Dublin, Ireland; Australian Catholic University, AUSTRALIA

## Abstract

Diabetic retinopathy (DR) is a prominent reason of blindness globally, which is a diagnostically challenging disease owing to the intricate process of its development and the human eye’s complexity, which consists of nearly forty connected components like the retina, iris, optic nerve, and so on. This study proposes a novel approach to the identification of DR employing methods such as synthetic data generation, K- Means Clustering-Based Binary Grey Wolf Optimizer (KCBGWO), and Fully Convolutional Encoder-Decoder Networks (FCEDN). This is achieved using Generative Adversarial Networks (GANs) to generate high-quality synthetic data and transfer learning for accurate feature extraction and classification, integrating these with Extreme Learning Machines (ELM). The substantial evaluation plan we have provided on the IDRiD dataset gives exceptional outcomes, where our proposed model gives 99.87% accuracy and 99.33% sensitivity, while its specificity is 99. 78%. This is why the outcomes of the presented study can be viewed as promising in terms of the further development of the proposed approach for DR diagnosis, as well as in creating a new reference point within the framework of medical image analysis and providing more effective and timely treatments.

## Introduction

The human eye is a complex organ consisting of almost forty interconnected elements, including the retina, iris, optic nerve, etc, and it can be affected by several diseases such as diabetic retinopathy (DR), glaucoma, cataracts and others. Among the long-term complications of diabetes, DR ranks high and is the second most common cause of blindness in the world, affecting the middle-aged and elderly. This is caused by damage to the tiny vessels that supply blood to the retina, which can develop slowly over time and cause blindness if not treated. A sedentary lifestyle that includes factors such as low physical activity and increased consumption of foods rich in fats and sugars has also contributed to the further spread of the disease. The WHO has estimated that about one billion people in the world are affected by disorders related to eyes. Currently, DR impacts roughly 537 million individuals globally in 2021, with estimations forecasting that the number of people affected will increase to 783 million by 2045 [1–3]. Such conditions must be diagnosed at their early stages and treated because if the retina worsens, it may lead to further complications [4]. DR has been grouped into various categories but ranges from no observable retinopathy to Proliferative Diabetic Retinopathy, where extensive damage of the retina is observed. In the first stage, termed Non-Proliferative Diabetic Retinopathy (NPDR), retinal damage can be observed, but there are no new vessels.

**Fig 1:**
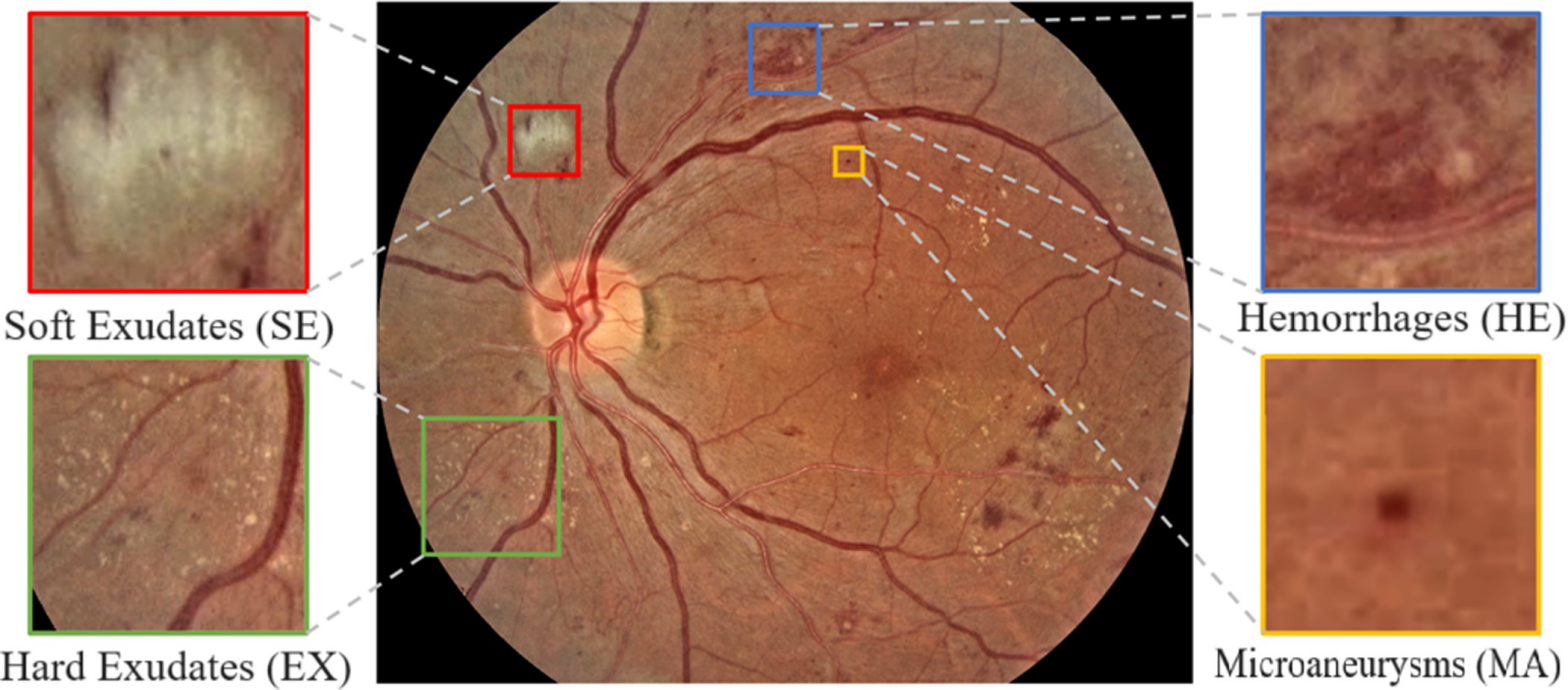
General DR lesions observed in the fundus images.

In contrast, in the Proliferative Diabetic Retinopathy (PDR), the retinal damage is severe and new vessels will have formed [5]. Fig 1 presents the typical signs of DR in a retinal fundus image: HE, SE, MA, and H. This detailed visualization also helps identify the extent of DR and, hence, the need for constant monitoring to combat this disabling condition [6].

**Fig 2:**
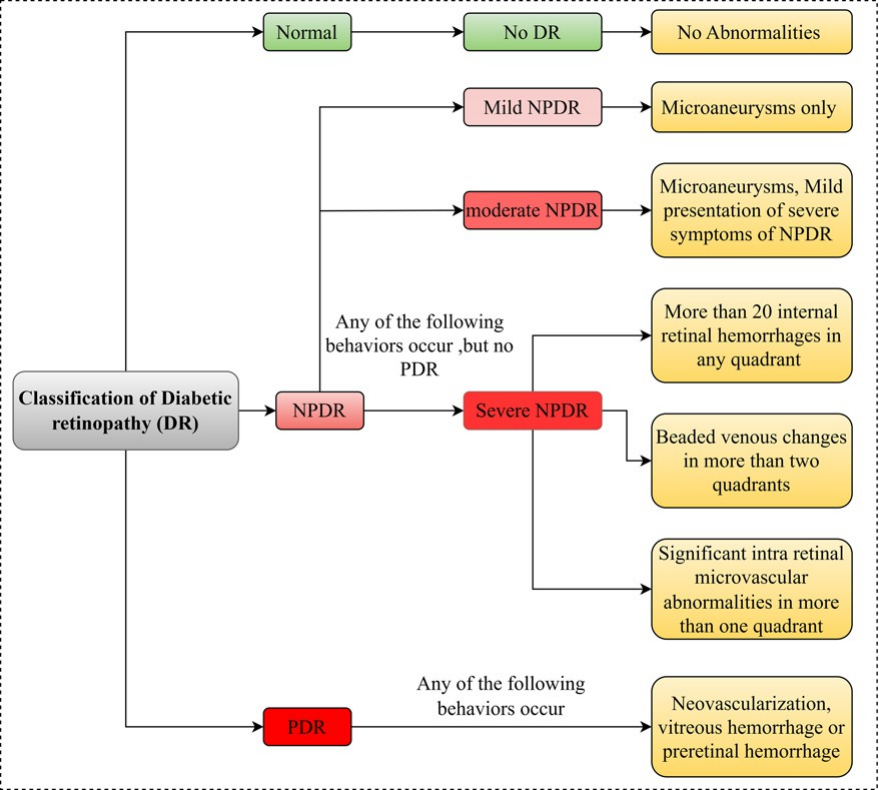
Stages of development of diabetic retinopathy.

The following is a brief outline of the development of DR: Fig 2 shows the stages where blood vessels narrow and later become blocked, and may result in complications such as microaneurysms, haemorrhages, and the formation of new blood vessels. This exemplification embraces both NPDR and PDR phases and, thus, illustrates the essential events that transpire at every stage of the disease.

The various factors and stages of DR make it crucial to monitor and accurately assess ophthalmic diseases regularly. Traditional diagnostic techniques face challenges in treating patients in complicated health cases, thus marking artificial intelligence’s significance in medical fields. Sophisticated algorithms, i.e. artificial neural networks and support vector machines [7], are employed frequently because of their ability to analyze complicated patterns of medical images such as DR where there is irregular growth of blood vessels [8–11]. The efficacy of these machine learning models depends on the quality and amount of medical data fed into them, underscoring the significance of acquiring good quality data to enhance the accuracy of diagnosis and condense the complications caused by diseases through early detection.

In addition, there have been advancements in the use of deep learning algorithms in medical diagnostics with the help of image segmentation. These techniques allow for predicting pixel-wise classes of the input images, which may help accurately segment the regions of interest needed to properly identify particular lesions in retinal images and other disease conditions or abnormal findings in medical imaging [12–14]. The development of Convolutional Neural Networks (CNNs) has ushered in great improvements in image processing technologies, improving the ability of automated feature detection and prediction. However, they are not ideal for segmentation tasks where pixel-level classification is needed because of their fully connected layers, which prioritise the class probability rather than the object’s structure [15]. Fully Convolutional Networks (FCNs) have been introduced to overcome these challenges, reducing the traditional CNN structures and substituting the fully connected layers with the convolution and de-convolution layers, thus improving the pixel-level segmentation speed [16, 17].On this basis, FCEDN introduces both the encoder and decoder components, greatly enhancing the segmentation performance since the feature maps of corresponding scales can be extracted and reconstructed to match the original image size in detail and at scale. Models such as FCEDN largely depend on the quality and amount of training data, often manually labelled by physicians. To reduce the problem of data restriction and improve the model’s robustness in unseen data, sophisticated data augmentation methods such as GANs are applied [18]. These models produce photorealistic images that help augment the training data and achieve better and more efficient diagnostic results in the healthcare sector [19–21].

This study presents a new method that complements the features of deep learning techniques, as seen in prior research, while rectifying their limitations on computational time and clinical relevance. In order to illustrate the advantages of our model, we compare it with the listed technologies and emphasize the increase in the speed of the calculations and decrease in the number of computations, and, at the same time, we highlight the possibility of achieving better precision in clinical diagnosis, especially in mammography image inspection.

The science of early diagnosis of the disease still has not grown to fully meet one of the biggest tasks of checking early signs of breast cancer. Such reliance on non-automatic results of mammograms means that the process requires the services of expert radiologists and special equipment, both of which are time-intensive and expensive. While this approach poses some demands of knowledge, it remains vulnerable to the variation of human discretion. Variability may also be the cause of rather substantial discrepancies. However, little ones may be insignificant, and on the other hand, big ones may lead to failure in recognizing early cancer signs or mistaking new findings for benign entities. It does this while offering potential health risks and, at the very least, could cause unnecessary patient stress. These problems demonstrate the present and urgent need to develop and establish more uniform and more applied methods for the interpretation of mammography. To address this need, the work employs complex computational tools to analyse the data to enhance the efficacy and reliability of mammography interpretations. We focus on cutting-edge CAD systems with deep learning and artificial intelligence applications. Modern CAD systems, as opposed to earlier ones, are more advanced and accurate in detecting minimal irregularities in mammography. Instead of conventional CAD technologies, we pay attention to those CAD technologies that are based on artificial intelligence. These innovations are much advanced compared to the methods used for radiological processes over many years [22–25]. However, deep learning, especially the convolutional neural network (CNN), has shown much improvement in image processing in the recent past, allowing tremendous improvements in automatic feature detection and enhanced classification [26].

Although CNNs are widely appreciated for their magnificent image classification performance, they have drawbacks when applied directly to segmentation problems. This shortcoming is associated with fully connected layers in standard CNNs that have to work harder and emphasise the inherent spatial architecture, which is important for correct pixel-level semantic segmentation to provide suboptimal results [27]. To address this problem, scholars then developed FCNs, which significantly shifted to the pixel-level segmentation by changing CNNs’ fully connected layers with the convoluted and deconvolution layers, which enhanced the optimisation of the segmentation processes [8, 15, 16, 28]. As for the design of FCNs, mainly because FCNs are not dense layers, this enables faster training sessions even with fewer parameters. A prototypical FCN includes a Convolution layer, a pooling layer, a rectified linear unit layer, and an un-pooling layer only at the end. However, as shown in the following sections, the FCN architecture is constrained by a non-trainable up-sampling layer, which limits its performance. Training these models with a diverse data set is essential, and GANs are valuable in this regard. Creating realistic medical images with GANs allows data sets for training deep learning models to be extended without exposing individual patients. This extends the model’s convenience in diagnosing different DR conditions in the other groups of patients.

Similarly, while segmentation has improved, knowledge sharing has been added to the deep structured learning toolbox. TL harnesses the benefits from existing abundant CNN models that can extract features from new limited datasets without learning the models from scratch [13, 29, 30]. The approach is useful when working with large-scale labelled data sets, where the features defined by the first layers of CNN can be used in other datasets. After the feature extraction step, the next step uses models such as Extreme Learning Machines (ELM). ELMs, at first developed for single-layer feed-forward networks, were later modified and implemented in deeper structures such as CNNs. These are far more effective in terms of learning curve than conventional deep learning architectures, guaranteeing high classification rates and avoiding deterioration [31]. When thinking about the general workflow for segmenting images and identifying specific areas of interest, one would start with FCNs for more precise pixel-level delineation. When the parameters of FCEDN have been adjusted to the specifications required, and when the training datasets have been enhanced with images generated by GANs, transfer learning methods may be utilized to extract pertinent features from these separated regions. Finally, by considering features, ELM-based classifiers can rapidly and effectively classify them while providing a one-stop solution to complex image processing problems. This research can potentially revolutionise the diagnosis of DR in its early stages. This new approach will establish new standards for objective and efficient medical image analysis in ophthalmology, and subsequently, contribute towards enhanced treatments and improved patient care.

### Research objectives

The need to fix the efficacy and performance of fundus image evaluation has formed the basis of research into improved methods. Despite being highly credible due to the involvement of experts, conventional manual evaluations of a student’s performance are tiresome and have inter and intra-rater reliability issues. To fill these gaps, this study proposes to use synthetically generated data together with segmentation models and state-of-the-art classification techniques to enhance the accuracy of FIs’ analysis. Particularly, a Generative Adversarial Network (GAN) is used for the synthetic image generation process, and a Fully Convolutional Encoder-Decoder Network (FCEDN) is chosen for segmentation. Therefore, transfer learning acts as a revolutionary step in which selection models are used to specify feature extraction. The assumption of Extreme Learning Machines (ELM) is backing this progress, which is known to classify information rapidly and accurately. It can be seen that the integration of transfer learning along with ELM in the analysis of machine-driven FIs can be a new, valuable discovery. This pressing requirement in the rapidly evolving medical imaging domain suggests that FIs interpretation should be improved. Adopting complex computational approaches may bring about the need for higher accuracy and faster DR detection. To this end, our research endeavours to derive concrete objectives that will guide the quest of developing an innovative and novel deep-learning framework optimised for perceptive mammogram evaluation. The main objectives of this study are as follows:

- To review the existing practices of traditional FIs interpretation methods as comprehensively as possible, paying most attention to the strengths and possible weaknesses of the existing methods.
- To train deep fusion algorithms for the DR dataset to distinguish between healthy and unhealthy systems, as the benign and malignant ROIs are similar, while the normal and tumour classes differ significantly.
- To achieve the best feature extraction, specifically in cases with limited data available, various novel methodologies should be incorporated into the FIs analysis process, such as a synthetic data generation technique and segmentation models.
- To perform an original classification modality to the trial and error to achieve fast and accurate categorization after the feature extraction phase.
- To compare the results obtained using the combined approach with those obtained when using the regular methods of analyzing mammogram images in terms of accuracy, sensitivity, specificity and time taken.
- To test the proposed methods’ scalability and modularity to include larger data sets and future developments in mammogram imaging technology or other data environments.

This study aims to enhance FIs analysis by incorporating synthetic data generation, segmentation models, and sophisticated classification methods to achieve these objectives.

### Key contributions of the research

Specifically, improving the analysis of the Fundus images is highly beneficial in the context of the constantly evolving medical imaging landscape. Diabetic retinopathy (DR) screening is the main critical issue in diabetes care that this study aims to solve by utilizing state-of-the-art computing. It attempts to propose and solve some of the key research problems found in the literature. The goal is to have an automatic retinal fundus image analysis system. Our goals provided the framework for an efficient, accurate, and robust analysis platform that will transform DR screening and identification. The main findings of the article are:

- Nature-Inspired Multi-Enhanced Method and KCBGWO Optimization: New methods for determining the severity of DR features have been proposed and put into practice. One such method is the K-Means Clustering-Based Binary Grey Wolf Optimization (KCBGWO) approach, which is used to fine-tune the parameters of the proposed FCEDN and ELM to improve the performance of segmentation and classification.
- DR-Net Framework Efficiency: Examined how well the DR-Net architecture performed while classifying Fundus pictures using various datasets for multiclass classification.
- Adaptive Histogram Equalization (AHE) and Synthetic Data Generation: Applied AHE to reduce noise and improve image quality in the early stages. Created synthetic pictures based on DR phases where data deficit was solved by transforming them into high-resolution fundus images using GANs, particularly GauGAN.
- FCEDN and Transfer Learning Hyper-Parameter Optimization: A hyper-parameter optimized FCEDN was used for semantic segmentation, improving pixel-by-pixel classification accuracy—an important part of early disease detection. We used transfer learning to extract features for our models, considerably reducing overfitting and training time.
- Fine-Tuning ELM and Extensive Performance Analysis: The final hidden layer of ELM was optimized for DR classifications, proving the model’s functionality. Contrasts the DR-Net with other cutting-edge techniques, demonstrating the DR-Net’s superior performance.
- Impact and Implications: highlighted the prospect of more tailored and targeted treatment plans as well as preventative measures for DR and highlighted the change in diagnosis as a significant development.

The work proposes novel deep learning networks as well as uses advanced GAN-based data augmentation methods to achieve the best results in automating and improving the accuracy of the diagnosis of DR. Adopting the KCBGWO algorithm to optimize the hyperparameters in FCEDN and ELM models is a step-up from previous methods, thus improving the models’ performance and making quality diagnostic tools available to different healthcare facilities.

The article’s outline is as follows: The literature review section unveils major developments in the past through a comprehensive analysis. The proposed research methodology section describes the techniques employed in this research, as described below, as well as the experimental results, and the discussion section explains the findings independently. Lastly, in the conclusion section, the authors provide a summary of the research and the implications resulting from the study.

## Literature review

In the medical imaging field, which is rapidly updating, generative models are crucial for extending the often poorly labelled datasets. These models not only enhance the data density of the model but also greatly enhance the diagnostic accuracy and effectiveness of the medical field. It remains common to refer to deep learning as artificial intelligence. Still, it is a subcategory of complex computing techniques that have become the healthcare sector’s foundation, particularly in precision medicine. It has been proven to work in various diseases like retinal diseases [12, 32–36], breast cancer [14, 37, 38], skin cancer [39], arrhythmia [40], Alzheimer’s disease [41], intracranial diseases [42, 43], HIV infections [44], as well as lung cancer [29, 30].

### Progresses in DR diagnosis

Some of the significant advancements include the development of a multitude of methods for DR diagnosis based on deep learning; as a result, numerous innovative methodologies have been proposed in the detection of DR. Later at the [45] the multi-scale shallow CNNs were implemented which outperformed all the existing models. Still, these models were very much dependent on the variations in input data. Based on this, [46] that can be seen as laying down some important advances in lesion detection. However, problems remained, especially with micro-aneurysm location because of the fluorescein dye. This has been a good move, as adopted ResNet50 and VGG16 were expeditious in detecting DR lesions. However, the identification of micro-aneurysms because of fluorescein was one of the obstacles that were encountered. To improve the accuracy of DR detection, a 3D-CNN ensemble was implemented. However, this decision was made without significant concerns about the features used [47]. In addition to the many layers, [48] used several types of CNNs; while this strategy offered richness to the computational process, it came with its computational costs.

Moving to the preprocessing, [49] used UM to improve the sensitivity but with the cost of losing image edges. Embracing the possibility of deep learning, [50] attempted the DR class prediction, outlining directions for improvement. [51] An initial attempt at early DR detection using only the dimensionality reduction methodology was remarkably innovative but at the expense of eradicating spatial information. This was adapted from famous architectures [52–54], which created a Siamese-like CNN structure that initially had reasonable levels of success. However, its effectiveness when transferring to other datasets is yet to be determined.

While attempting to establish a high level of exudate detection [55], the researchers obtained fairly good results, but training took a long time. The DeepDR framework originally presented by [56] performed exceptionally well vis-à-vis its specificity and sensitivity, although the algorithm’s resilience when operating within more diverse sets merits a look into. Concerns regarding the uneven Gray Levels manifested minor drawbacks to the CNN approach [57]. Moving to the preprocessing models, [58] tested some potential but were flattered with noisy pictures. For instance, the ML classification model was flexible with [59] but posed maintenance issues. Building upon prior work that aimed to refine classification techniques, the [60] approach obtained satisfactory VTDR grading accuracy. VTDR risk detection was the focus of both [61] and [62], where the need to improve model’s performance and the dataset used were highlighted. [63] The CNN technique, which used a two-stage CNN approach, looked quite successful but also required more processing resources.

### Advancements in synthetic image generation and segmentation

Furthermore, deep learning (DL) has assumed the important role of image synthetic data generation in segmenting retinal disease. For example, [64] used Pix2Pix architecture [65] to produce high-resolution synthetic multi-parametric MRI brain images. This process starts with establishing normal brain segmentation maps extracted from T1-weighted scans, followed by a series of linear transformations that add tumour labels to the maps. These improvements result in the creation of high-resolution synthetic MRI images. While extending the usage of generative models, [66] employing GANs to synthesize fake liver lesion ROIs for improving the CNN classification of complex lesions, including cysts, metastases, and hemangiomas. They proposed using three types of DC-GANs, each trained for a specific lesion type, and an AC-GAN that supervised all three lesions altogether [67]. It was also clear that the DC-GAN models performed better than the AC-GAN ones and offered a higher sensitivity and specificity than the basic CNN improvements.

Building on the benefits of generative models, the researchers produced artificial chest X-ray pictures using a DC-GAN [19]. The efficacy of the CNN in identifying disorders, including infiltration, atelectasis, and unremarkable finds on the NIH ChestX-ray14 [68] dataset, was improved by incorporating these generated pictures into the actual images. Incorporating both synthetic and real data significantly increased the classification accuracy and verified the effectiveness of the proposed approach in leveraging synthetic data to enrich actual datasets. [69]used a CycleGAN [70] uniquely to create NCCT from CECT images, improving the capacity to assess the severity of the illness. This shift made the improvement of the segmentation of essential organs including the kidneys, liver, and spleen possible. In addition to streamlining the imaging procedure, the method boosted the Dice scores significantly from 0.535 to 0.747. This underscores the significance of synthetic scans in augmenting the training set and the possibility of noteworthy progress in diagnosis.

Generative models are beneficial in cases where there is a scarcity of images available for a certain ailment, such as Diabetic Retinopathy (DR). [20] introduced DR-GAN, a sophisticated multi-scale U-Net-like network specifically developed for generating high-resolution fundus pictures by leveraging information related to DR stages and lesions information. The synthetic images were utilized in future tasks, like DR grading and lesion segmentation, to enhance the accuracy of the well-known EyePACS [71] and the FGADR [72] dataset. The study [73] employed a two-step methodology, where the initial part utilized ProGAN to construct semantic label maps of the retina arteries. The maps were transformed into realistic retinal pictures using the image-to-image translation network. This approach was extensively trained and verified on the DRIVE and CHASE_DB1 datasets, and it achieved segmentation accuracy that was either comparable to or superior to the current leading methods.

### Feature selection and optimization techniques

In the topic of segmentation, several investigations have been carried out; one prominent pioneer is [74]. They used convolutional neural networks (CNNs) to understand the retinal images and the U-Net model to identify the optic disc in VTDR. They concentrated on employing morphological filtering and watershed modification for the detection of OD (Object Detection) and OC (Object Counting) [75]. The exudate detection method used Deep and convolutional networks with SVM classifiers [76]. However, in their investigation of glaucoma optic neuropathy screening, the authors [28] and [77] proposed an ensemble system that effectively integrated both the local and global image levels. The approaches vary with existing methods proposed by Ferreira et al. (2018), Ran et al. (2018), Zhang et al. (2017) and Xu et al. (2020) by proposing segmentation and classification methods for cataract detection. Li et al. extended the previously stated technique by introducing models [76–78] that employ more intricate frameworks.GoogleNet-CAM is utilized to conduct automatic cataract detection. The significance of hyperparameter selection in deep learning networks has been emphasized in [79–81]. In order to address the complex nature of manual tuning, scientists have proposed applying biomimicry-inspired techniques such as quantum-behaved PSO [82] and particle swarm optimization (PSO) [83]. The grey wolf optimization (GWO) algorithm [86] aims to replicate the hunting habits of grey wolves. However, other variations of the Grey Wolf Optimizer (GWO) have been suggested. The primary issue is that the early GWO population is expected to lack a clear direction, as highlighted in [84–88]. This lack of direction may subsequently impact the algorithm’s effectiveness.

### Research gap

Among the major challenges identified, one of them is that deep learning models are very sensitive to the input data and variations in them can have a huge effect on the precision and training of the models. However, using advanced models, including deep convolutional neural networks, presents a problem of high computational requirements for a model that may not always be feasible to implement in every clinical setting. Even with all these advancements, there is an issue of segmentation precision, particularly in images with different contrasts and conditions, which greatly affects diagnostic accuracy. The use of Convolutional Neural Networks also presents another major problem, which is the lack of large datasets marked with particular medical conditions such as Diabetes Retinopathy has an impact on how machine learning models are trained. In addition, it would be challenging to consistently get the optimal model performance since deep learning network hyperparameter tweaking is time-consuming and sensitive to human intervention.

Our research proposes a variety of innovative approaches to overcoming these issues:

- Enhanced Synthetic Data Generation: We use cutting-edge Generative Adversarial Networks (GANs) to produce artificial pictures that are more lifelike and better represent the pathological circumstances of the patient. This method trains deep learning models more effectively and becomes more generalizable to other medical problems.
- Optimization Algorithms: They also apply nature-mimicry algorithms such as the Grey Wolf Optimization (GWO) to enable the automation and enhancement of hyperparameter selection. This strategy helps minimize manual tuning and, therefore, improves the efficiency and preciseness of the model training process.
- Advanced Segmentation Techniques: Therefore, the suggested technique seeks to improve the pixel-wise classification performance by utilizing Fully Convolutional Networks (FCNs) and Fully Convolutional Encoder-Decoder Networks (FCEDN). These models are specifically designed to function with medical pictures, yielding segmentation results that are more precise and crisp.
- Utilization of Extreme Learning Machines (ELM): The ELMs are incorporated to improve the detection and classification function after the segmentation stage. As mentioned earlier, these ELMs have a fast learning ability, making them suitable for real-time diagnosis of medical ailments and can easily accommodate large data sets.

It is anticipated that the specific problems identified in the literature will be addressed by these suggested solutions, which will also raise the bar for medical image analysis by increasing its efficiency, speed, and adaptability. While substantial progress has been made, the primary unresolved challenges in the field include the lack of large annotated datasets, computational complexity, and the manual effort required for hyperparameter tuning. Our model addresses these concerns by leveraging synthetic data generation, advanced optimization algorithms, and enhanced segmentation techniques to improve overall performance in medical image recognition.

## Proposed research methodology

**Fig 3:**
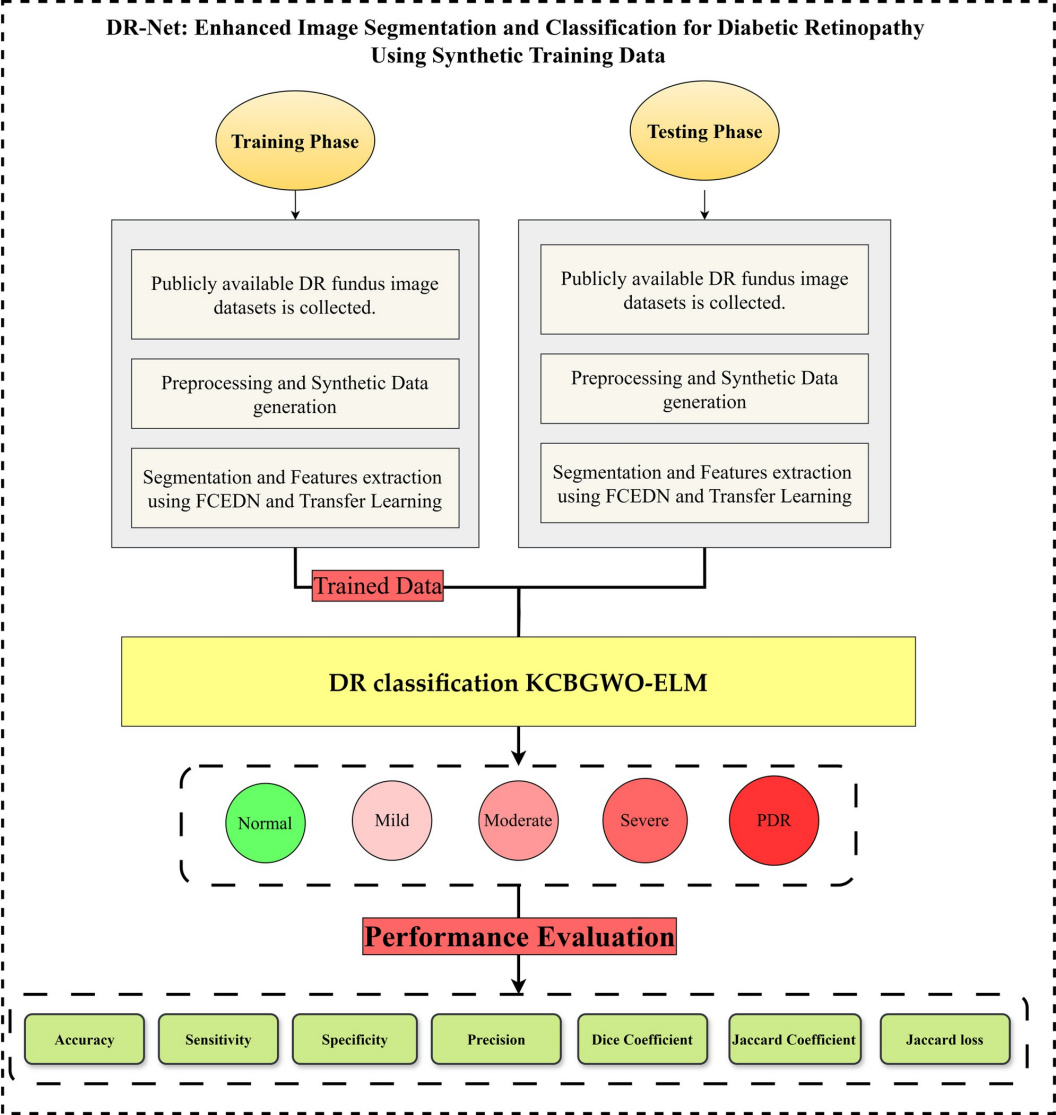
Proposed research methodology for DR diagnosis.

The study presents a new method for DR that applies multi-class classification to the DR-Net architecture. A block diagram that depicts the steps involved in DR detection is shown in Fig 3. when using the proposed approach. This diagram clearly illustrates how the proposed system efficiently identifies and categorizes DR using DR-Net.

### Dataset

**Fig 4:**
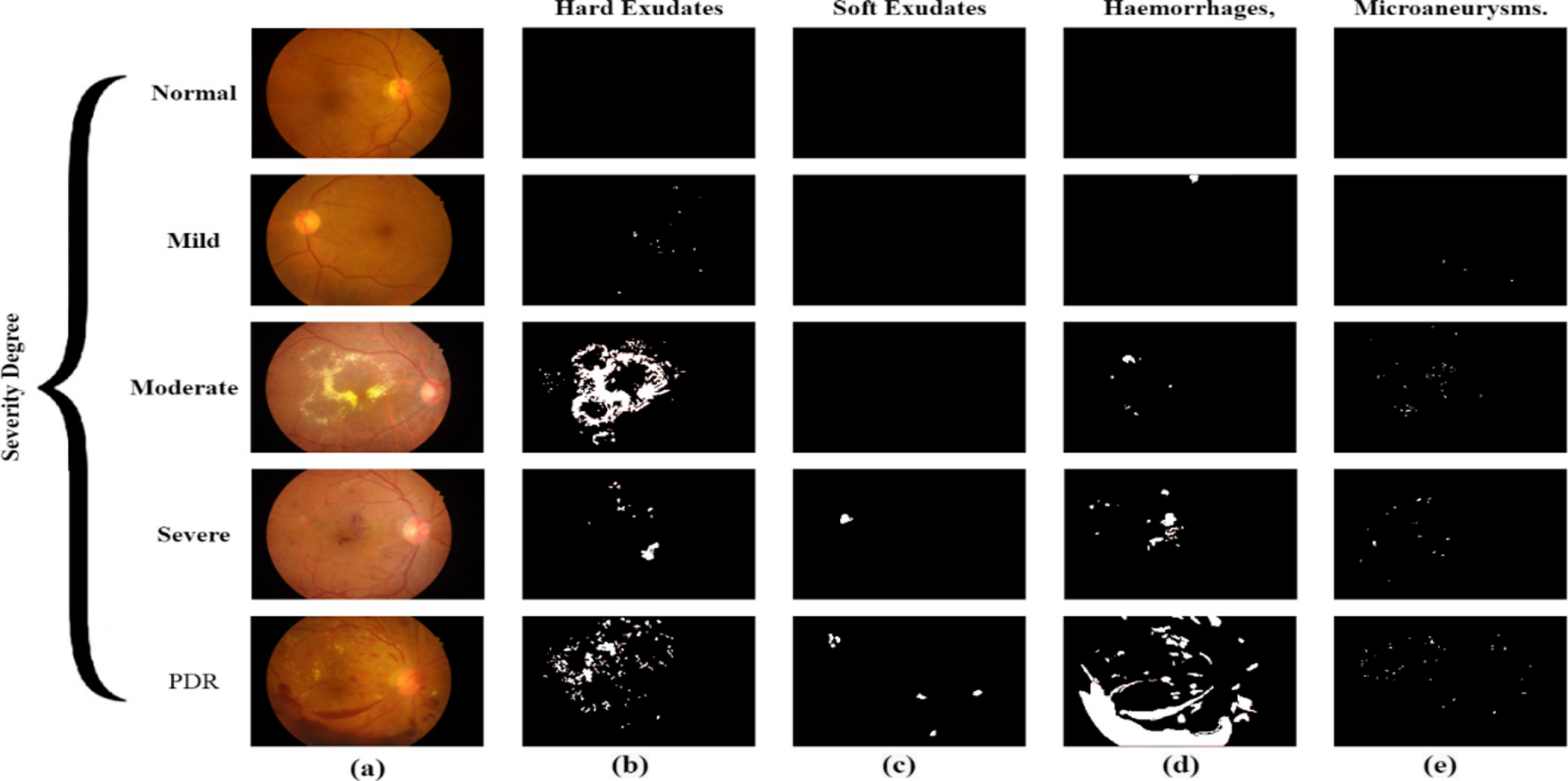
(a) Retinal Lesions in Fundus Images, (b) hard exudates, (c) soft exudates, (d) haemorrhages, and (e) microaneurysms.

The open public dataset Indian Diabetic Retinopathy Image Dataset (IDRID) [89] is used to identify macular oedema and DR from retinal images employing the computerized technique. In this way, this dataset can be viewed as a useful source of information for researchers who focus on developing computer-assisted screening and diagnostic means. IDRID encompass high quality, colour fundus images: IDRID encompasses high quality, colour fundus images:

Regarding the photographs, retinal signs for both DR and macular oedema were labelled, including microaneurysms, haemorrhages and both hard and soft exudates. Such annotations encompass various progressive phases of the disease, including the stages of mild and proliferative DR. IDRID provides retinal images with high resolutions of 4288 x 2848 and 3456 x 2304 pixels; this is critical in grading the disease since features that may be of significance are well spotted. All the photographs are catalogued by professionals who can illustrate the disease’s presence and degree. The IDRID dataset has 134 images without DR, 20 images with MNDR, 84 with M NDPDR, 74 with S NDPDR, and 49 with PDR. In addition, a subset refers to instances of DME, but as a count, it differs from the other subcategories because some cases may belong to them. The segmentation masks in this dataset are designed to achieve precise spatial accuracy for four types of lesions: Soft exudates, hard exudates, haemorrhages, and microaneurysms. As shown in Fig 4, the images included in the IDRID dataset are fundus images (FIs) with their ground truth masks. This dataset is mostly used for modelling purposes to train models, which are used to detect and predict the severity of DR and diabetic macular oedema. Experts utilized this for the final tuning of models with such tasks as transfer learning, data augmentation, and the new state-of-the-art convolutional neural network architectures. This is important as it offers better and well-annotated data sets for improving the field of medical image analysis and, in turn, the patients’ care due to early diagnosis of diseases with high accuracy.

### Retinal fundus images fusion using GANs

**Fig 5:**
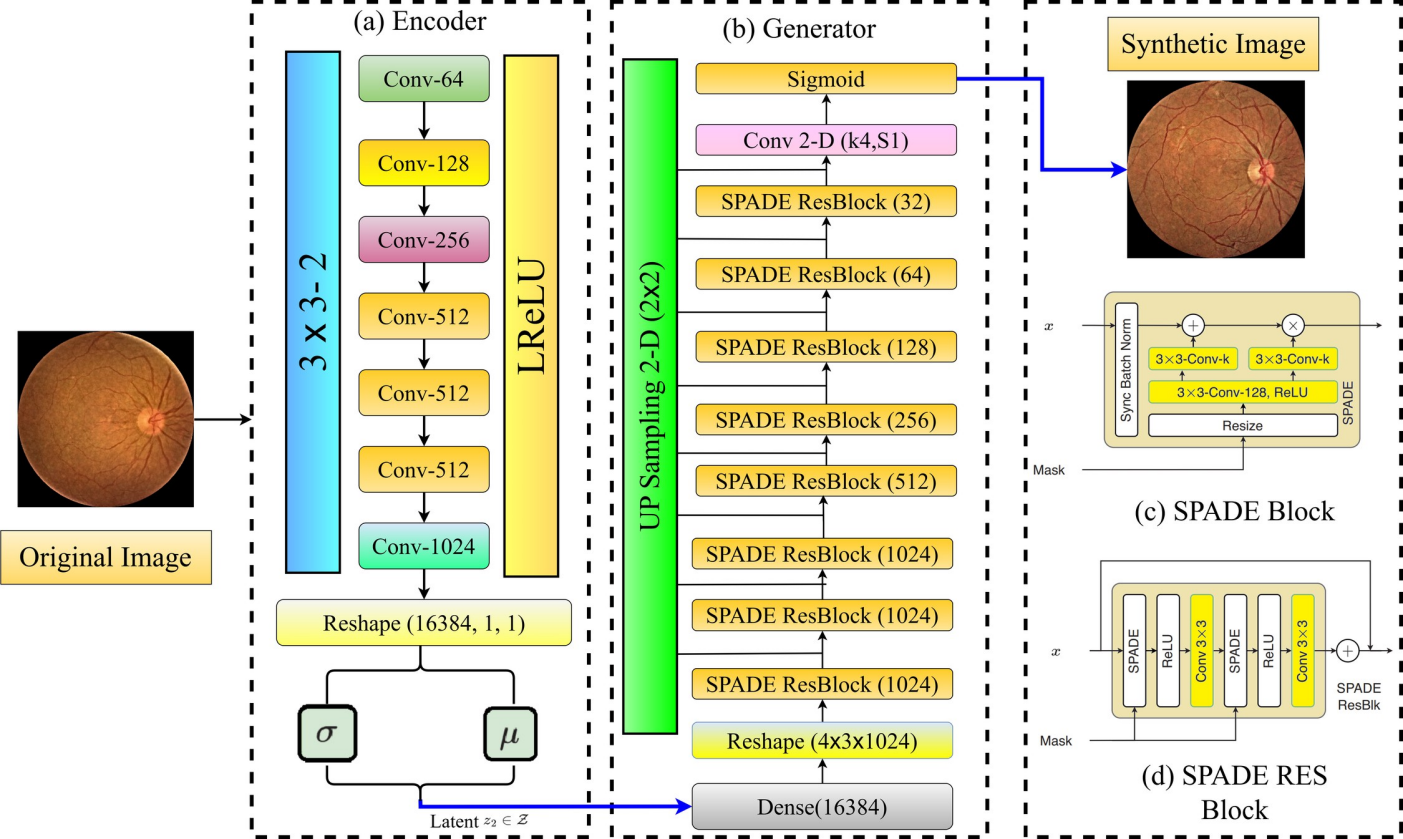
GauGAN architecture.

The fundus images are reconstructed using an algorithm known as GauGAN to produce realistic fundus images. GauGAN is based on Generative Adversarial Networks (GANs), VAE-GANs, and class-conditioned Variational Autoencoder (VAE). This structure allows GauGAN to steer the Generator with a style, as shown in Fig 5, which depicts the entire structure during training.

The Encoder is shown in Fig 5(A), which uses the fundus pictures as input to obtain the mean and variance of a Gaussian distribution. Fig 5(B) illustrates how the Generator employs a residual learning architecture. From a Gaussian distribution, it generates the retina fundus pictures using one-hot encoded semantic lesion maps and randomly chosen latent vectors, z2 ∈ Z. Adding stochasticity improves the pictures’ diversity by using a variational sampling strategy. Features from the mask used as a conditioning factor can be easily integrated with the help of the SPADE (SPatially-ADaptivE normalization) approach. For each semantic label map, SPADE effectively learns unique scaling and bias parameters, as shown in Fig 5(C), hence maximizing the spatial adaptation of these parameters. GauGAN uses four loss functions to train the Generator, while one is used for the Discriminator. The first step in training the Generator is calculating the GAN loss, which is determined by dividing the expectation by the Discriminator’s predictions:

$$L_{G}=-{\mathbb{N}}_{z∼Pz},y∼Pdata\;[D(G(x),)]$$ (1)

The Generator utilizes Feature Matching Loss to synchronize the feature spaces of the Generator and the Discriminator with the original images, effectively reducing discrepancies in the Discriminator’s assessments of both original and generated images. The loss formula is expressed as:

$$L_{F}=\|(D((y)-(G(x),y)))\|1$$ (2)

Moreover, VGG Feature-Matching Loss is implemented to ensure that the synthetic images closely resemble the real images used in pre-training on ImageNet. This involves comparing the feature map outputs from various layers of a pre-trained VGG-19 model, specifically relu 1_1, relu 2_1, relu 3_1, relu 4_1, and relu 5_1. The corresponding loss function is:

$$L_{V}=-\mathbb{N}.E_{z∼P_{z},y∼P_{data\;}}\sum_{i=1}^{5}\frac{1}{2i}\left[\left\|VGG\left(\left(y,M_{i}\right)-\left(G(x),M_{i}\right)\right)\right\|1\right]$$ (3)

The Encoder adopts a KL-loss function to ensure the latent vectors adhere to a normal distribution. This is defined as:

$$L_{K}=D_{K.L}(q(z|w)\|p(z))$$ (4)

The total loss for the Generator, which combines these components, is calculated as:

$$L_{G}=L_{F}+L_{V}+L_{K}$$ (5)

This strategy ensures a balanced optimization across different dimensions of the generative model.

### Binary Grey Wolf Optimizer based on K-means clustering-based

**Fig 6:**
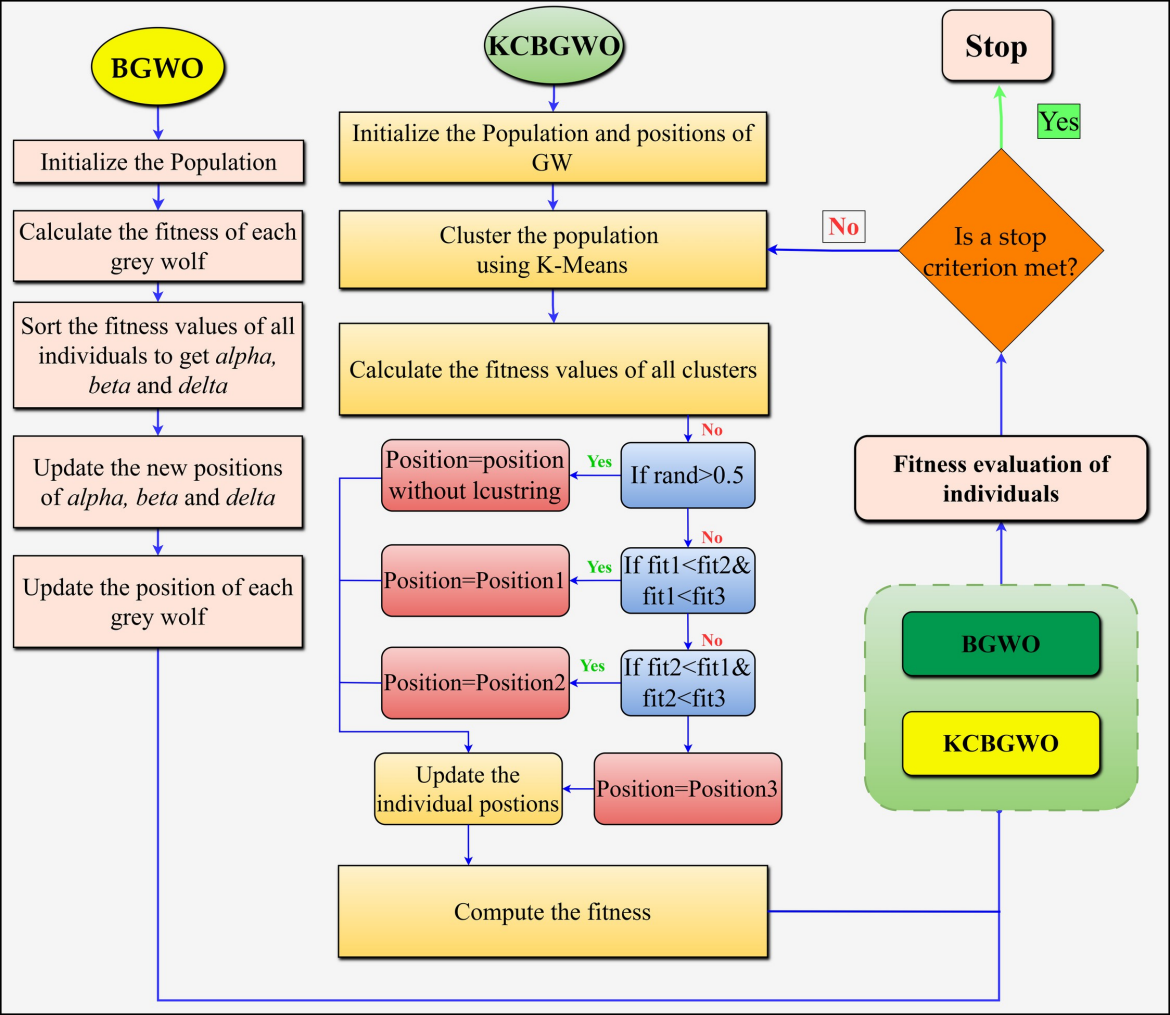
KCBGWO flowchart.

As depicted in Fig 6, KCBGWO integrates Binary Grey Wolf Optimizer (BGWO) with K-means clustering, enhancing the optimization process by effectively navigating the search space. The initial population P consists of N grey wolves, each represented by a binary vector. $x_{d}^{i}$Initialized randomly:

$$X_{i}^{d}=\left\{\begin{array}{c} 1\\ 0 \end{array}\;with\;probability\;0.5\right.$$ (6)

This random initialization ensures a diverse population that aids in avoiding local optima. The population is divided into KKK clusters via K-means clustering:

$$P=\cup_{i=1}^{K}\;K_{i}$$ (7)

The cluster K_*i*_ is epitomized by its centroid K_*i*_, calculated as:

$$K_{i}=\frac{1}{\left|K_{i}\right|}\sum_{X_{j}\in K_{i}}X_{j}$$ (8)

The centroids help update the search process by guiding wolves towards better solutions. The objective function minimizes the sum of squared distances within clusters:

$$Minimize\;\sum_{i=1}^{k}\sum_{j=1}^{k_{i}}{\left\|X_{j}-K_{i}\right\|}^{2}$$ (9)

Wolves are ranked by fitness *f*(*X*_*i*.*j*_), and roles (α,β,δ,ω) are assigned to the most fit:

$$f\left(X_{i.j}\right)\;for\;j=1,2,\ldots ,\left|K_{i}\right|$$ (10)

Wolves are given specific roles, which include α, β, δ, and ω. The ranking assures:

f(α)≤f(β)≤f(δ)≤f(ω) (11)

The hunting behaviour of grey wolves inspires the position update. The distance between wolves and prey is calculated using:

$$K=\left|C\cdot X_{p}(t)-X(t)\right|$$ (12)

Here, A and C vectors are updated to control exploration and exploitation:

$$A=2ar_{1}-a$$ (13)

$$C=2r_{2}$$ (14)

These vectors help balance randomization and convergence. The updated position of the wolves is given by.

The updated position of the wolf is as follows:

$$X(t+1)=X_{p}(t)-A.K$$ (15)

The green arrow, representing the ideal result, shows how the equation rearranges each wolf’s location to get them closer to the prey. The step size, determined by the distance from the prey and the caliber of the best solutions, is defined by the word A.K. This updating strategy is akin to how grey wolves hunt, which entails shifting their positions to get closer to their target. The dynamic adjustment ensures that the wolves continuously refine their locations while they hunt for answers, enhancing the quality of the answers.

The sigmoid function is utilized to adjust the continuous dynamics to binary search spaces:

$$\sigma (x)=\frac{1}{1+e^{-x}}$$ (16)

The updated rule definition of the binary position is:

$$x_{d}^{\left(t+1\right)}=\left\{\begin{array}{cc} 1 & \;if\;\sigma \left(\frac{x_{d}^{1}+x_{d}^{2}+x_{d}^{3}}{3}\right)\\ 0 & \;othjrewise\; \end{array}\geq \right.\;rand$$ (17)

This allows smooth transitions between continuous and binary spaces, improving the optimization of binary decision problems. Each wolf’s fitness is evaluated using a problem-specific function. A problem-specific fitness function f is used to evaluate each wolf on an individual basis:

$$f\left(X_{i}\right)=Fitness\left(F_{i}\right)$$ (18)

The KCBGWO algorithm minimizes the Euclidean distance between data points and centroids, ensuring local search efficiency. The parameter *a*(τ) linearly decreases over iterations, transitioning the search from global exploration to local exploitation:

$$a(\tau )=2-\frac{2\tau }{T}$$ (19)

Finally, the optimization process stops when the maximum number of iterations *T* is reached or when the fitness of the alpha wolf meets a predefined threshold.


$$\begin{array}{l} f(\alpha )\;then\;\alpha =F_{i}\\ \;If\;f\left(F_{i}\right)<f(\beta )\;and\;f\left(F_{i}\right)\geq f(\alpha )\;then\;\beta =F_{i}\\ f(\delta )\;and\;f\left(F_{i}\right)\geq f(\beta )\;then\;\delta =F_{i} \end{array}$$ (20)

Finally, the optimization process stops when the maximum number of iterations *T* is reached or when the fitness of the alpha wolf meets a predefined threshold.


StopIfτ≥Torf(α)reachesapredefinedthreslhold (21)

The KCBGWO algorithm is a powerful hyperparameter optimization tool, particularly effective for binary and categorical search spaces. Integrating clustering and binary updates balances exploration and exploitation, offering machine learning experts an efficient method for optimizing algorithm configurations. The pseudocode for this algorithm is presented in **Algorithm 1**, providing a step-by-step guide for implementation.

### Algorithm 1. Pseudocode of KCBGWO

Input:
N ‐ Total number of wolves (Population size)
 C ‐ Clusters count
 D ‐ Dimensions of the search-space
 T ‐ Max number of iterations
 f–Fitness-evaluation function
Output:
 α ‐ Optimal solution discovered
Procedure:
1. Initialize Population:
 Create a population (ρ) with n wolves, each with a D-dimensional binary vector.
 For each wolf: *F*_*i*_ *in ρ*
  For each dimension d = 1 to D:
   *F*_*i*_[d] = GenerateBinary()
2. Form Clusters:
 Apply K-means to split ρ into K clusters.
 For each cluster *K*_*i*_:
  Calculate its centroid using the average position of wolves in C_i.
3. Assign Roles:
 For each cluster C_i:
  Order wolves by their fitness using f.
  Designate roles: α (Alpha), β (Beta), δ (Delta), ω (Omega) based on fitness order.
4. Update Positions:
 For iterations t = 1 to T:
  Adjust control parameter a:
   a = 2 * (1 - τ / T)
  For each wolf *F*_*i*_ *in ρ*:
   For top roles *F*_*p*_ in {α, β, δ}:
    Set vectors A and C:
    A = 2 * a * GenerateVector() ‐ a
    C = 2 * GenerateVector()
    Compute distance K from *F*_*ρ*_ *to F*_*i*_:
    K = |C * position of *F*_*ρ*_ at τ ‐ position of *F*_*i*_ at τ
    Update position for the next iteration:
    *F*_*i*_[τ+1] = position of *F*_*ρ*_ at τ ‐ A * K
    Use a sigmoid for binary transitions:
    For each dimension d = 1 to D:
     probability = Sigmoid((X_α.[d] + X_β.[d] + X_δ.[d]) / 3)
     *F*_*i*_[d][t+1] = 1 if GenerateRandom() < probability else 0
  Reassess fitness and readjust roles:
   For each wolf *F*_*i*_ in P:
    Update α, β, δ based on new fitness evaluations.
  Reapply K-means to adjust clusters and centroids.
5. Finalize:
 Return the α with the best fitness.
Supporting Functions:
GenerateBinary() ‐ Outputs 0 or 1 randomly.
GenerateVector() ‐ Produces a vector with random elements each in [0, 1].
GenerateRandom() ‐ Yields a random float between 0 and 1.
Sigmoid(x) ‐ Computes 1 / (1 + exp(-x)).

### Enhancing semantic segmentation with FCEDN and KCBGWO

CNNs are effective in classification tasks in computer vision, but their performance for segmentation is limited by their fully connected layers, which ignore spatial information. This leads to poor performance where pixel-level details are required, as the context is not captured. Since the fully connected layers are substituted by convolutional (Conv) and deconvolutional (de-Conv) layers, Fully Convolutional Networks (FCNs) are best suited to address the challenges. With this modification, FCNs may provide pixel-level outputs while maintaining spatial context—a crucial feature for picture segmentation.

FCNs use two main architectures for semantic segmentation: Two main architectures are used by FCNs for semantic segmentation:

Basic FCN Structure: Includes convolutional layer, rectified linear unit, pooling and up-sampling layer. While the Conv and pooling layers help to decrease image size and increase the abstraction of important features, the up-sampling layers bring the image back to its original size. However, upsampling without training the weights for the upsampling process is a limitation that may cause loss of details.

Advanced Encoder-Decoder Setup: The Advanced Encoder-Decoder Configuration in this study incorporates an encoder that conducts downsampling, coupled with a decoder that uses Transpose Convolution (TC) and Upsampling (UP) layers for precise upsampling. Incorporating trainable parameters during the upsampling phases is crucial for enhancing the network’s ability to replicate segmentation outputs with high precision and accuracy.

**Fig 7:**
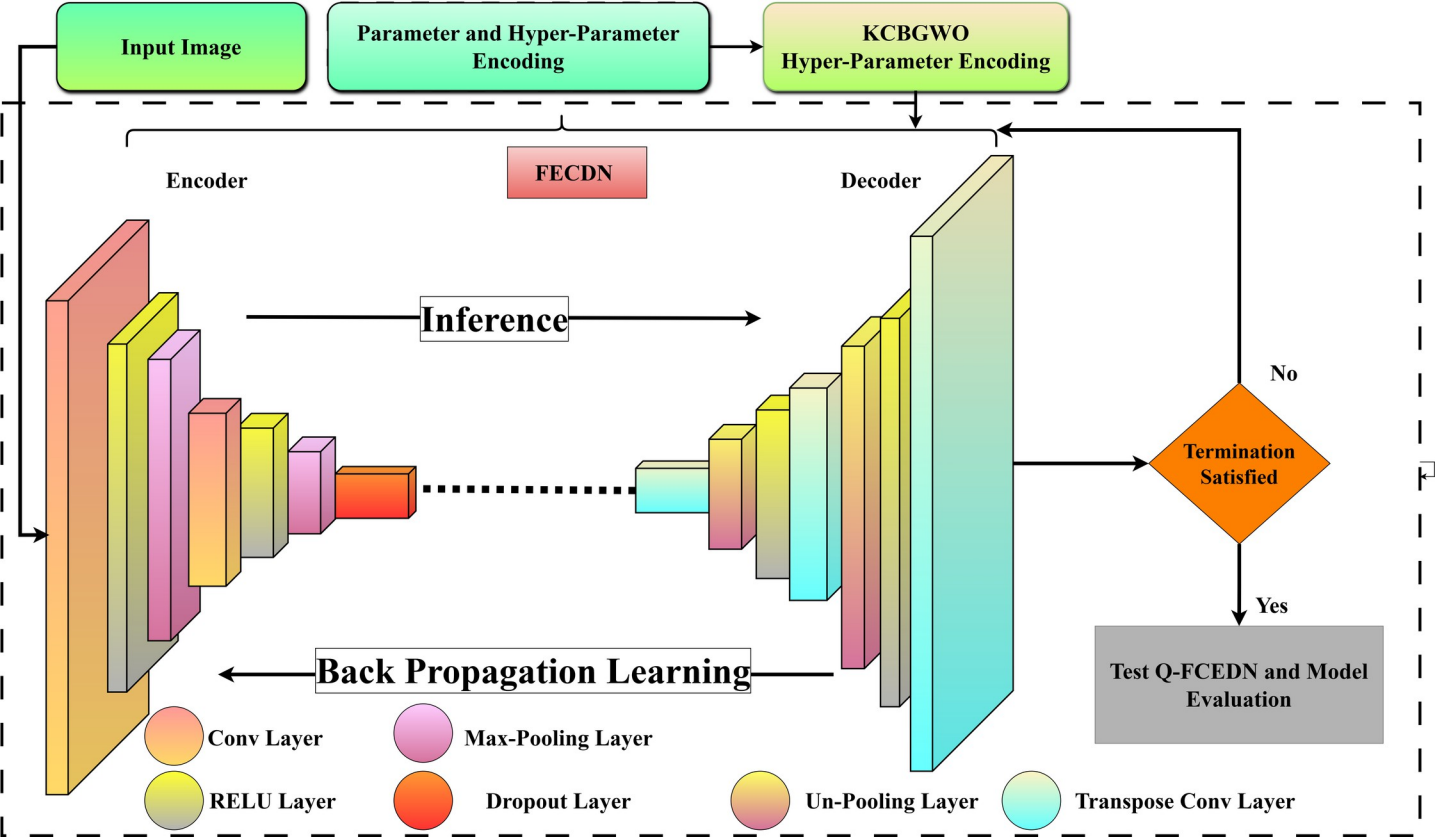
FCEDN architecture.

This Fully Convolutional Encoder-Decoder Network (FCEDN), illustrated in Fig 7, utilizes this sophisticated architecture. The encoder integrates convolutional layers, dropout, and max-pooling for efficient downsampling and feature extraction, reducing spatial resolution effectively. The decoder sequentially employs TC, UP, and additional dropout layers to upscale these features to the input’s original dimensions for precise segmentation. Fine-tuning the hyperparameters of the FCEDN is essential, with the K-Means Clustering-Based Binary Grey Wolf Optimizer (KCBGWO) playing a pivotal role in this process, encompassing four critical stages.

Encoding: Hyperparameters are thus represented in the Euclidean space of k dimensions, with each dimension representing the settings of each layer in the FCEDN.

Population Initialization: A number of potential solution vectors or ‘wolves’ are created, each a hyperparameter vector of different dimensionality.

Fitness Evaluation: Every vector is then analyzed to evaluate how much it enhances the Jaccard coefficient that measures the similarity between predicted and actual segmentations.

Population Update: The set of hyperparameters is improved through performance; the better ones are used to proceed to the next stages.

Besides, this approach helps to tune the FCEDN for the best performance and demonstrates the integration of highly sophisticated neural network designs with cutting-edge optimization algorithms. The end product is a reliable system that can achieve semantic segmentation at high levels of accuracy, which is suitable for various applications that require detailed examination of the spatial distribution of features, for instance, in medical imaging applications.

### Feature extraction

The use of deep learning, and in particular Convolutional Neural Networks (CNNs), has expanded significantly in domains such as text analysis, object identification, picture interpretation, and facial recognition. Convolutional layers, pooling layers, activation layers, dropout layers (if applicable), and fully linked layers make up the CNN models of the current generation. This work uses one of the most popular methods, employing CNN models that have already been trained to extract features from DR datasets. Therefore, the model can extract general characteristics from the fundamental convolution layers by using DR datasets, which can facilitate the model’s use in medical image analysis.

We chose and altered four well-known CNN models in order to modify CNN architectures for DR image datasets: VGG16, ResNet50, DenseNet201, and InceptionResNetV2 are among the models. These networks have two primary functions: Adjusting and extracting features. The technique is divided into two phases: feature extraction and fine-tuning. During the feature extraction process, the pre-trained model uses its past knowledge to identify pertinent characteristics in the newly collected data. This data is then fed into a newly trained classifier. Multiple convolutional layers extract various aspects from the input images: higher levels detect abstract and representational properties, mid layers identify textural and form characteristics, and low layers detect minor elements like edges and colours.

This research examines the similarities, contrasts, and potential for integration among these variables, considering the wide variety of DR picture types seen in our sample and compared with other datasets. Specifically, by investigating the feature vector fusion method from multiple pre-trained at different levels, we expect that merging features from the numerous models and the various levels would result in a better and more complete representation.

Several fusion techniques have been devised and examined to test these theories. This approach uses all the tangled layers from the various pre-trained models to extract feaand fuse features. Using only three models, doing away with InceptionResNetV2, and relying solely on the partial convolutional layers, Strategy 2 is comparable to Strategy 1. The final classification technique uses the information in the convolution layers close to the network’s bottom. Finally, but just as importantly, Strategy 4 combines the outputs of the final three ResNet50 blocks, mixing them into feature vectors using maximum pooling to get a more thorough feature representation.

**Fig 8:**
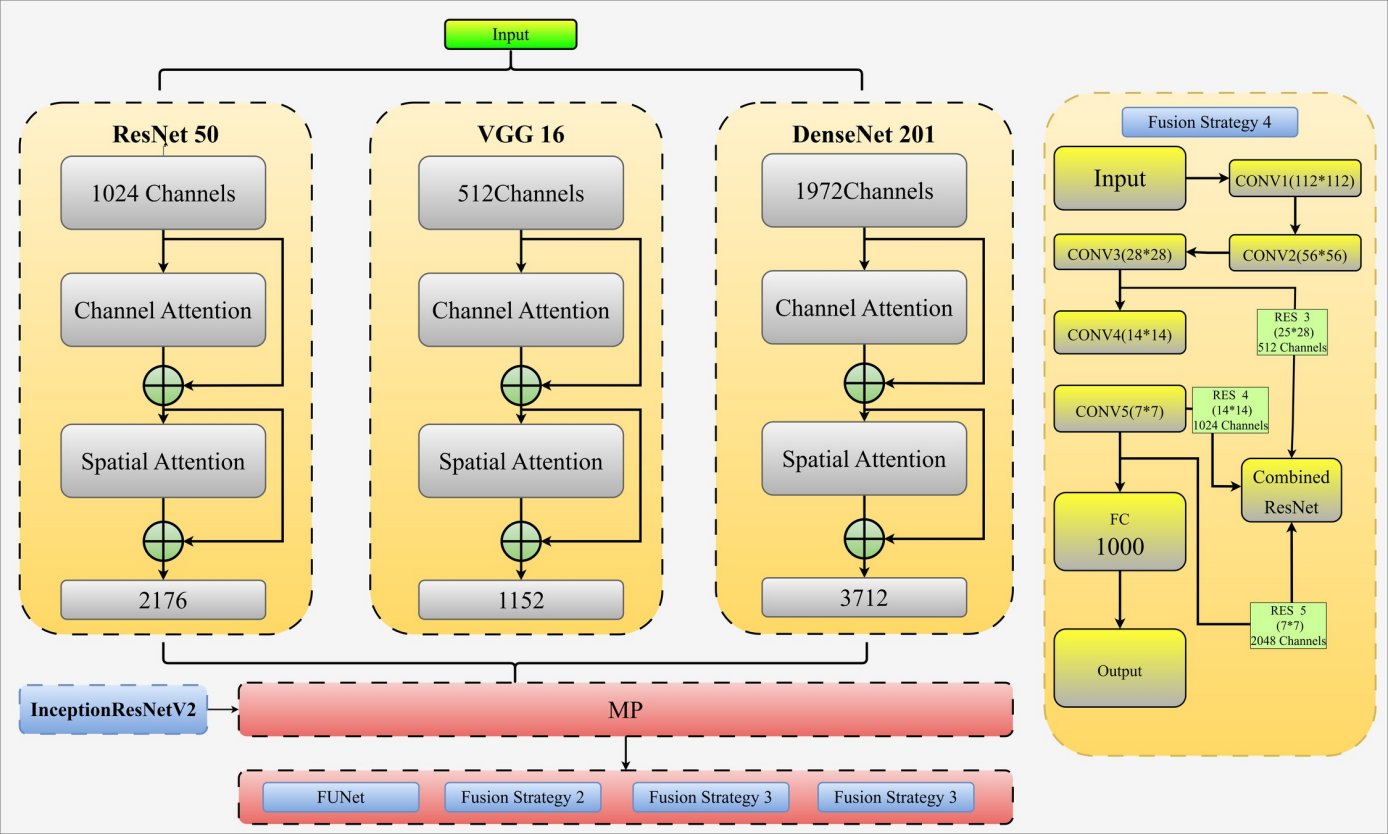
Overview of feature extraction with fusion approaches and the FuNet model.

Furthermore, an attention mechanism, an additional component frequently used in semantic segmentation and picture classification, was considered to comprehend subtle aspects. In order to minimize interference from other channels and preserve as much information as possible from various places while considering the relevance of distinct spaces, the FuNet model integrates channel and spatial attention modules. Fig 8 shows the specifics of the FuNet model’s Channel Attention and Spatial Attention organization and structure.

### KCBGWO-ELM-based DR classification using

**Fig 9:**
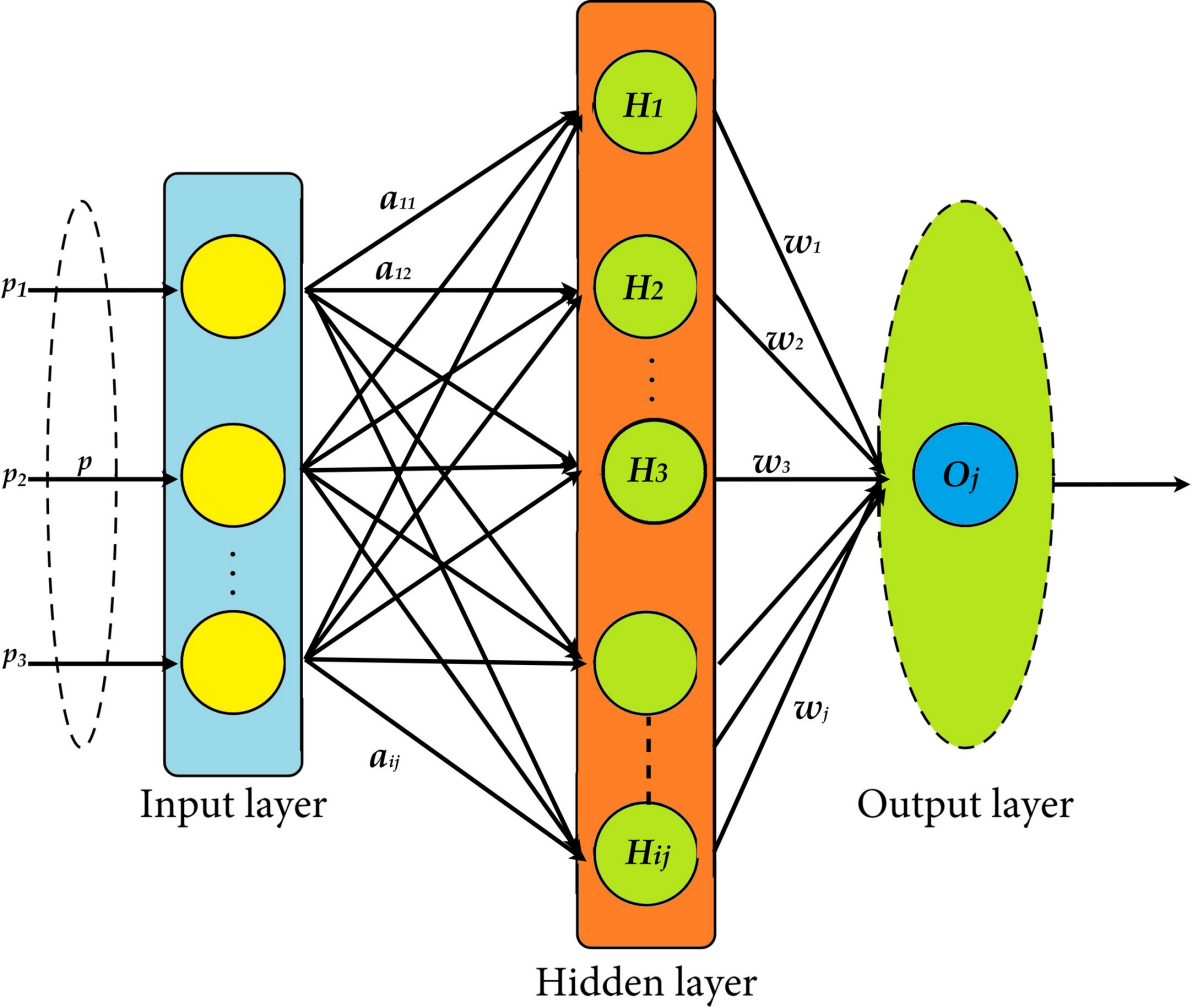
ELM configuration.

The study also proposes a novel technique for DR diagnosis using KCBGWO-ELM. The ELM [90] is a feed-forward neural network for different computational purposes, including classification, regression, and clustering. The back propagation algorithm that can be used with ELM includes single and multiple hidden layers, but what differentiates it is the static nature of the number of hidden nodes and their parameters which are biases and weights. In contrast to conventional backpropagation approaches and other algorithms that train neural networks that require frequent weight updates and can get trapped in local minima, the ELM parameters can remain constant or be used as supplied. The ELM algorithm works for minimum training error normalized weights for better performance, and some techniques are used to manage local optimum. In Fig 9, you see how the ELM works with the arrows pointing at each step. As for the fast learning ability, ELM usually performs better than those using backpropagation.

For the Single-Hidden Layer Feed-forward Neural Networks (SLFN) with G hidden-nodes and activation function f(x) defined, let a set of H-specific random sample points be defined as (*pi*, *t*_*i*_), where *p*_*i*_ = [*p*_*i*1_, *p*_*i*2_, …, *p*_*in*_] ^*T*^ ∈ *Q*^*n*^ And *t*_*i*_ = [*t*_*i*1_, *t*_*i*2_, …, *t*_*im*_] ^*T*^ ∈ *Q*^*m*^.

The underlying equation that governs this network is given as follows:

$$\sum_{i=1}^{G}w_{i}f\left(a_{i}p_{j}+c_{i}\right)=o_{j},\;\text{for}\;j=1,2,\ldots .H$$ (22)

The weight vector *a*_*i*_ = [*a*_*i*1_, *a*_*i*2_, …, *a*_*in*_] ^*T*^ establishes a connection between the ith hidden node and the input nodes, whereas *w*_*i*_ = [*w*_*i*1_, *w*_*i*2_, …, *w*_*in*_] ^*T*^ linked with the i^th^ hidden node to the output node. The variable *c*_*i*_ indicates the threshold value related to ^ith^ hidden node and *o*_*j*_ = [*o*_*j*1_, *o*_*j*2_, …, *o*_*jn*_] ^*T*^ depicts vector outputs for the j^th^ node generated by SLFN.

SLFNs with G hidden nodes and activation function f(x) can reliably predict a set of H samples with zero (0) error. The equation represents the level of accuracy:

$$\sum_{j=1}^{G}\left\|o_{j}-t_{j}\right\|=0$$ (23)

The equation, as previously mentioned, can be succinctly expressed as follows:

Where

M.w=T (24)

$$M\left(a_{1},\ldots ,a_{G},c_{1},\ldots ,c_{G},y_{1},\ldots ,y_{G}\right)={\left[\begin{array}{ccc} f\left(a_{1}\cdot y_{1}+c_{1}\right) & \cdots & f\left(a_{G}\cdot y_{1}+c_{G}\right)\\ \vdots & \ddots & \vdots \\ f\left(a_{1}\cdot y_{H}+c_{1}\right) & \cdots & f\left(a_{G}\cdot y_{H}+c_{G}\right) \end{array}\right]}_{H\times G}$$ (25)

$$w={\left[\begin{array}{c} w_{1}^{T}\\ \cdot \\ \cdot \\ \cdot \\ w_{N}^{T} \end{array}\right]}_{G\times n}$$ (26)

$$T={\left[\begin{array}{c} t_{1}^{T}\\ \vdots \\ t_{N}^{T} \end{array}\right]}_{G\times n}$$ (27)

The output matrix from the hidden layer is denoted by "M". Each column of matrix M represents the output produced by the kth hidden node in response to the inputs. y1, y2, and subsequent values up to yH. The solution to the linear system can be represented as:

$$w=M^{-1}.T$$ (28)

In this context, *M*^−1^ represents the Moore-Penrose generalized inverse of the matrix M. The output function of the ELM is defined as:

The ELM’s output function is defined as:

$$g(y)=p(y)w=p(y)M^{-1}T$$ (29)

#### KCBGWO-ELM

The KCBGWO-ELM model, which successfully classifies DR by combining the FuNet architecture with ELM and KCBGWO, is a unique model introduced in this paper. The following is a concise expression of the suggested model:

In order to extract the relevant characteristics, the DR dataset was first inserted into the FuNet architecture.

Configuring parameters: The population size, the maximum number of iterations, and the number of hidden layers for the ELM were then determined as control parameters. First, biases for the hidden layer and random input weights between -1 and 1 were applied to the population.

GWO Initialization: The vector’s constituent parts which hold the input weights for every possible Grey Wolf Optimizer (GWO) solution—are defined in this step. These parameters are used in the process of optimization.

Fitness evaluation: In order to evaluate the calibre of solutions produced at each optimization process iteration, a cost function is used. The fitness function employed was the Mean Square Error (MSE), which was computed as follows:

$$\mathrm{MSE}=(1/\text{n})*\Sigma \left[{\left(\text{oi}-\text{ti}\right)}^{\wedge }2\right]$$ (30)

The variables oi, ti, and n represent the observed values, anticipated values, and sample size.

Optimization Progression: The genetic binary grey wolf leader and follower people’s roles were adjusted and changed as the algorithm developed.

Parameter storage involved updating the weights and biases and saving the ideal values when the maximum number of iterations was reached. The procedure was restarted from the GWO startup stage if the maximum was not reached.

Training and Evaluation: The obtained optimized weights and biases were utilized to train the ELM classifier with the training set. Optimization was applied to create the solution vector suggested to reach optimal outcomes. Thus, it was evaluated whether the model fits the data and the possibility of its generalization with the help of the designated test set.

In this paper, we proposed and developed the KCBGWO-ELM method that combines feature extraction, parameter optimization, and classification, which presents a powerful solution to segment and classify images of DR.

### Performance evaluation

Evaluating the performance of models in classification tasks is crucial for applying machine learning in practice. This study uses a group of measures suitable for classification analysis and consists of diverse metrics. The following metrics are utilized to assess the model’s picture classification capabilities:

Accuracy (Ac): The proportion of the model’s correct predictions.

Sensitivity (Sn): This reflects the model’s ability to correctly classify positive instances.

Specificity (Sp): Overall, the ability to predict negative instances accurately.

Precision (Pr): The reliability of the leaders’ positive predictions.

In this way, by including all these measures presented in Table 1, we provide a more thorough assessment of the models segmentation and classification ability, facilitate further adjustments, and prepare the model for deployment in real-life environments.

**Table 1:** Mathematical representation of performance evaluation matrices.

Matric Name	Mathematical Representation
Ac	$\frac{\text{True}\;\text{Positives}+\text{True}\;\text{Negatives}}{\text{True}\;\text{Positives}+\text{True}\;\text{Negatives}+\text{False}\;\text{Positives}+\text{False}\;\text{Negatives}}\times 100\%$
Sn	$\frac{\text{True}\;\text{Positives}}{\text{True}\;\text{Positives}+\text{False}\;\text{Negatives}}\times 100\%$
Sp	$\frac{\text{True}\;\text{Negatives}}{\text{True}\;\text{Negatives}+\text{False}\;\text{Positives}}\times 100\%$
Pr	$\frac{\text{True}\;\text{Positives}}{\text{True}\;\text{Positives}+\text{False}\;\text{Positives}}\times 100\%$

## Experimental results and discussion

This section provides the quantitative results of the experiments conducted on the proposed experimental setup using the IDRiD dataset, particularly with the FIs data, to verify the efficiency of the proposed DR Classification method. MATLAB with GPU acceleration was used in the performance of the experiments for the study’s proposed algorithm. The above experiments were performed on the system with intel core i7 13th generation with 32 GB RAM and GeForce RTX 3090 Ti GPU. In order to measure performance, the IDsRID database, which is well-liked in the related field, was employed. The method they suggested for the enhancement of the current technique of power system load forecasting was the BGWO, which was modified by integration of K-means clustering and a weighting factor for the comparison with other algorithms and the existing methods such as the BGWO, the GWO, and the PSO. The above algorithms were implemented in MATLAB language on a high-end computer. The above operation was tested 30 times for each of the algorithms to make the comparison more realistic. The population was initialized to 30, and the maximum number of iterations was fixed to 500 for all the algorithms. Table 2 summarises the key hyperparameters and performance metrics for the BGWO algorithm integrated with K-means clustering and ELM.

**Table 2:** Hyperparameters and performance metrics of BGWO with K-means clustering.

	Population Size	Max Iterations (BGWO)	K-means Clusters (K)	Max Iterations (K-means)	Objective Function (Domain)	Best Iteration	Best Fitness Value	Weighting Factor α	Weighting Factor β	ELM Hidden Layers	ELM Input Weights Range
1	30	500	3	100	[0, 1]	12	0.0150	0.3	0.4	1	[–1, 1]
2						27	0.0320	0.5	0.3		
3						34	0.1100	0.4	0.3		
4						10	0.0330	0.2	0.5		
5						28	0.0320	0.3	0.4		
6						8	0.0900	0.5	0.2		
7						58	0.0910	0.6	0.5		
8						8	0.0150	0.2	0.4		
9						36	0.0330	0.4	0.3		
10						39	0.0320	0.5	0.6		
Avg						26	0.0553	0.4	0.4		

### Comparative analysis of KCBGWO with baseline methods

This paper evaluates the KCBGWO’s performance and compares it to well-developed metaheuristic algorithms, including the BGWO, GWO, and PSO. To assess the effectiveness of our proposed algorithm, we conducted evaluations using ten established benchmark functions. These functions are split into two categories: unimodal and multimodal. The unimodal group includes Sphere (F1), Schwefel (F2), Schwefel (F3), Schwefel (F4), and Generalized Rosenbrock (F5). For the multimodal category, we analyzed Generalized Schwefel (F6), Rastrigin (F7), Ackley (F8), Griewank (F9), and Generalized Penalized (F10). Each algorithm was analyzed according to the methodological approaches outlined in the original papers on their development. Table 3, which enumerates the performance of the algorithms, shows that KCBGWO yields the best performance compared to the other algorithms for most of the test functions. The results of each function and their specific performance metrics presented in the results table emphasis the effectiveness of KCBGWO in these assessments. As for the separate algorithms, as it could be observed, the BGWO, GWO, and PSO were able to perform specific functions effectively, whereas KCBGWO outperformed them or was more competitive in most of the tasks. Subsequent visualizations like that captured in the expected graph will further show the convergence patterns of these algorithms. These visuals are expected to demonstrate that the KCBGWO algorithm is proficient in managing the trade-off between exploration and exploitation throughout the early and later phases of the optimization procedure.

**Table 3:** Optimization algorithms performance comparison across benchmark functions.

	KCBGWO		GWO		PSO		GA	
Function	Av	Std	Av	Std	Av	Std	Av	Std
**f1**	2.01e-26	6.02e-26	4.97e-18	8.01e-18	6.97e+00	1.67e-15	9.64e+02	3.14e+02
**f2**	2.44e-15	1.67e-15	1.34e-11	1.07e-11	2.09e+00	4.34e-16	7.34e+00	2.34e+00
**f3**	1.34e+00	4.84e+00	1.81e+01	2.49e+01	3.89e+03	8.89e-13	4.04e+04	7.64e+03
**f4**	6.34e-05	8.14e-05	3.79e-02	4.94e-02	2.09e+01	3.44e-15	5.14e+01	1.19e+01
**f5**	2.69e+01	6.49e-01	2.79e+01	5.64e-01	3.99e+03	8.99e-13	1.16e+05	8.19e+04
**f6**	4.24e+03	1.04e+03	4.89e+03	1.17e+03	5.84e+03	2.64e-12	1.08e+04	2.59e+02
**f7**	1.37e+01	1.54e+01	2.64e+01	3.34e+01	6.49e+01	1.34e-14	1.74e+01	1.34e+00
**f8**	1.01e-13	7.54e-14	3.44e-10	2.54e-10	3.74e+00	1.27e-15	5.84e+00	7.64e-01
**f9**	2.64e-03	1.47e-02	1.47e-02	4.39e-02	1.59e+00	4.34e-16	8.44e+01	2.54e+01
**f10**	1.64e+00	1.37e+00	3.04e+00	1.29e+00	1.99e+02	2.79e-14	1.44e+02	8.39e+01

### Data balancing and segmentation results

This paper aims to understand GAN sampling and how it can be used in balancing datasets. The training set provides further information about the imbalance in the data, characterized by the condition that the number of instances in the most frequent class, grade 3, is six times higher than in the least frequent class, grade 0. Thus, the following steps are proposed: To overcome this, GAN sampling was applied in order to generate fake samples for each class so that all classes have the same number of samples as the class with the most samples. Another 25 synthetic images were generated to complement existing examples and make the classes more balanced regarding the presence of GAN-derived samples.

The process was, therefore, one where retina fundus images were generated from lesion maps depicting the DR grades ranging from 0 to 4. Lesion maps in RGB representation were synthesized according to the DR grade. GauGAN was employed to generate synthetic fundus images; thus, the study presented practical examples of utilizing modern image synthesis techniques in the medical field. The image processing starts by performing binarization of the image to enhance the low-intensity regions, which are likely to contain red lesions, converting them into bright white shapes on a black background. Morphological techniques are used to fine-tune these shapes by filling gaps and depicting potential lesions as compact formations to exclude black specks that represent non-essential white noise. Each outlined region is examined for geometric features, including the lesion size, complexity of the borders, and elongation, to distinguish the real red lesion from image artefacts.

**Fig 10:**
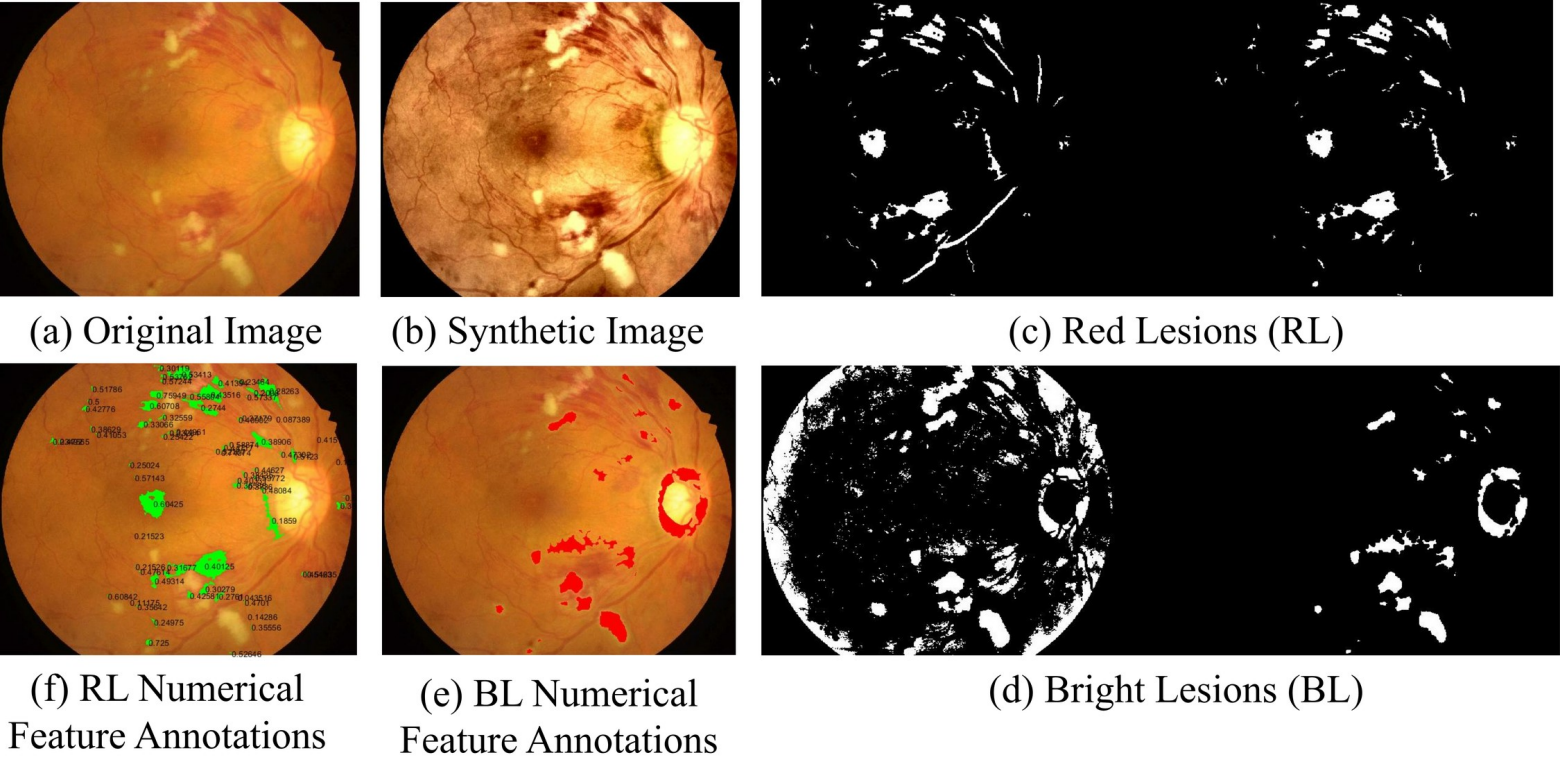
Detailed portrayal of the diabetic retinopathy identification workflow. (a) Initial retinal image, (b) Enhanced image post-preprocessing, (c) Identification of red lesions (RL), (d) Identification of bright lesions (BL), (e) Quantitative feature labels on bright lesions, and (f) Quantitative feature labels on red lesions.

For bright lesions, the approach is slightly different but follows the same steps as above, changing the binarisation threshold to highlight the image’s lighter parts. Possible bright lesions are also analyzed through morphological changes and geometrical factors to ensure that false bright areas or other reflections are not mistaken for true lesions. The process ends with Fig 10, which demonstrates the segmentation results and provides an overview of the DR diagnosis process’s detailed and complex series of actions. Subfigure (a) depicts the true retinal image, subfigure (b) provides a synthetic image, and subfigure (c) indicates the detected red lesions (RL) of the retina, which are microaneurysms or haemorrhages, marked as Figure d shows the bright lesions (BL) as detected from the image segmentation process, which is the exudates or cotton wool spots. Subfigure (e) superimposes numerical feature descriptions of the bright lesions’ shape, size, and other measurements. Similarly, arrows and numbers in Subfigure (f) represent the red lesions in terms of numerical values, offering a precise quantitative evaluation of the identified lesions.

### Classification results

This paper focuses on a complex, innovative approach to the feature extraction based on the GoogLeNet model used in computer vision tasks. The development of the KCBGWO-ELM is the main purpose of this research, and its effectiveness will be verified in this study. Because of such limitations as memory constraints, it became necessary to down-sample by incorporating specifications for the maximum pooling layers in the feature output layer. We obtained a set of CNN-based feature combinations, designated K1 through K4, to obtain a complete picture. Specifically:

- K1, which is based on ResNet50 and uses the full potential of the convolutional layers, includes 2048 features.
- The K2 feature is from the DenseNet201 model, including its entire convolutional structure and 1920 features.
- K3, obtained from the full convolutional basis of the VGG16 model, contains 512 features.
- K4, K4 uses the entire convolutional base of InceptionResNetV2, amounting to 1536 features.

We also found three feature sets—L1, L2, and L3—extracted from the same models that concentrated on particular intermediate layers:

- L1 comprises 1024 distinctive characteristics harvested from the ’conv4_block6_out’ layer within the ResNet50 architecture.
- L2 captures 1792 features sourced from the ’conv4_block6_out’ layer of the DenseNet201 network.
- L3 secures 512 features from the ’block4_pool’ layer associated with the VGG16 model.

Further expanding the array of feature sets, M1, M2, and M3 have been introduced, each sourced from specific intermediate layers unique to their respective models:

- M1amasses 512 features extracted from the ’conv3_block4_out’ layer in the ResNet50 framework.
- M2 gathers 512 features from the ’conv3_block12_concat’ layer found in DenseNet201.
- M3 pulls 256 characteristics from the ’block3_pool’ layer of the VGG16 model.

These enhanced feature collections provide a robust framework for deep analytical applications.The following explains where the sets of features are derived from, how they are extracted, and their dimensionality to help with any tasks involving images and classification.

**Fig 11:**
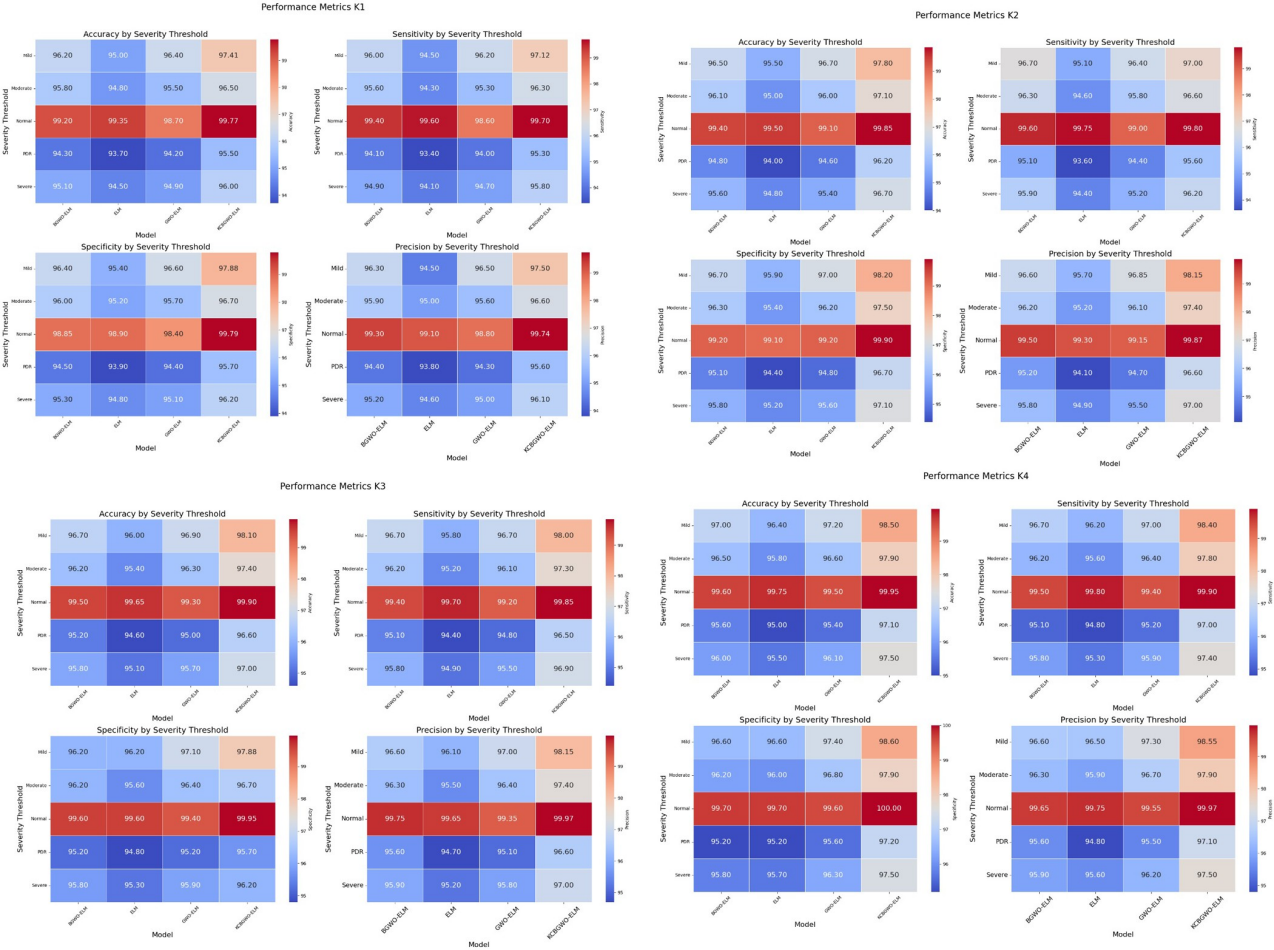
Feature extraction-based classification results using pre-trained CNN models.

The study’s heatmaps show the performance metrics of four sophisticated models: For five severity levels Normal, Mild, Moderate, Severe, and PDR—the performance of various forms of ELM, GWO-ELM, BGWO-ELM, and KCBGWO-ELM is compared across four categories, K1, K2, K3, and K4. Each heatmap depicts important metrics: Four individuals are: Precision, the fraction of total examples for which the model produced accurate predictions; An indicator of sensitivity is the proportion of correct results to the total number of results (both positive and negative); Precision: the proportion of correct negative results to the total of both positive and negative results; Precision: the percentage of correct positive predictions out of all the cases that the model classified as positive. In Category K1, ELM has proved to have high accuracy for the Normal severity threshold of 99. 35% but declines as the severity rises, having 93%. 70% for PDR. On the other hand, the proposed model KCBGWO-ELM has relatively higher accuracy, consistently outperforming other models in all severity thresholds with accuracy for a normal of 99. 77% and for PDR 95. 50%. The sensitivity for ELM starts at 99. normality is at 60% and decreases to 93%, confirming the negative effect of mental health on normality at 40% for PDR. On the other hand, KCBGWO-ELM achieves high sensitivity for Normal 99. 70%, and for PDR 95. 30%. Sensitivity for ELM varied with different studies ranging from 98%. 90% (Normal) to 93. 90% (PDR), while KCBGWO-ELM, ranging between 99, achieve the highest values. Normal range 79% to 95%. 70% for PDR. As for the measure of precision, it is slightly lower for ELM and equals 99. 10% (Normal) to 93. For non- Normal data, 80% (PDR), KCBGWO-ELM has high accuracy for Normal 99. 74% and for PDR 95. 60%. Similar trends are observed in Categories K2, K3, and K4, where KCBGWO-ELM outperforms all other models in all evaluated metrics. Fig 11(A) portrays K1, which is a comparison.

For Category K2, the ELM model generally achieves accuracies ranging from 99% to 100%, with a notable exception where the accuracy drops to 98%. The recognition accuracy for typical algorithms is quite low, often around 0% for PDR, whereas the KCBGWO-ELM model demonstrates substantially higher accuracy, achieving 99.85% for Normal and 96.20% for PDR categories. ELM’s sensitivity remains high at 99.75% for Normal and 93.60% for PDR. In contrast, KCBGWO-ELM shows an even higher sensitivity, reaching 99.80% for Normal and 96.50% for PDR. Accuracies for ELM typically span from 99% to 100.10% in the Normal category and 94.40% in PDR, with the lowest false positive rates among the models examined. KCBGWO-ELM outperforms others with the highest specificity scores of 99.90% for Normal and 96.70% for PDR. The precision of ELM varies from 99.30% in Normal to a lower range in PDR, notably 94.10%. For KCBGWO-ELM, precision rates are even higher, with 99.97% in Normal and 96.60% in PDR. The accuracy of muscle units (MUs) identified in Category K3 for ELM oscillates between 99.65% in Normal and 94% in PDR. Several methods, such as PIR-ELM (99.21%), KCE-ELM (98.34%), KCC-ELM (98.94%), KCF-ELM (99.45%), and KCBGWO-ELM (99.71%) show varied effectiveness. The sensitivity for ELM in this category is also high, noted at 99.70% for Normal and 94.40% for PDR, with KCBGWO-ELM achieving even higher rates. In Category K4, ELM starts at a base accuracy of 99.75% for Normal and goes up to 95% for PDR, whereas KCBGWO-ELM peaks with 99.95% for Normal and 97.10% for PDR. The specificity of ELM spans from 99.70% in Normal to 95% in PDR, and for KCBGWO-ELM, it reaches 100% in Normal and 97.20% in PDR. These models are depicted in Fig 11(B)–11(D), allowing for a clear comparison of their performance under various conditions. The enhanced performance of KCBGWO-ELM is visually represented in bar graphs, with darker colours indicating higher performance metrics.

The discussion below outlines various methodologies to improve integrating features from multiple data sources. One effective approach involves applying preprocessing techniques, which transform the data into a format better suited for integration. These methods ensure that the disparate data types are harmonized, enabling more seamless and effective analysis. This paper also discusses other strategies to facilitate FS 1, a fusion strategy combining feature vectors from K1, K2, K3 and K4, leading to a combined vector with a feature-length of 6016. Fusion Strategy 2 (FS 2) combine the features from L1, L2 and L3, and the feature vector length becomes 3328. The Fusion Strategy 3 (FS 3) combines the feature vectors of M1, M2, and M3, which generates a vector of dimension 1280. Res 3 includes features from Res 2 and the previous FS and results in a vector of 2304; Res 4 follows the same pattern concerning Res 3 and has a vector of 2664; Res 5 takes the features from Res 4 and produces a final vector of 3584.

Moreover, the FuNet model also uses a particular fusion technique that combines features from three component models denoted as L1, L2, and L3; the feature extent is augmented here to 7040 through attention blocks. These strategies are significant for the extraction of various and rich characteristic from input images and, therefore, achieving image classification. The research employs different PMs with unique layers to identify important features from the DR image domain. To optimize the fusion strategies implemented above, these were carried out carefully to maximise the classification models’ performance and accuracy. The paper also compares the accuracy of several Machine learning algorithms such as ELM, GWO-ELM, BGWO-ELM, and the proposed KCBGWO-ELM for different stages of DR, including normal, mild, moderate, severe, and proliferative (PDR) stages. Furthermore, the FuNet model is evaluated, concretely comparing it to conventional approaches to demonstrate the advantages of modern fusion techniques in improving diagnostic outcomes depicted in Table 4.

**Table 4:** Classification outcomes utilizing feature extraction from diverse pre-trained CNN models and various down sampling approaches.

Classifier	Class	AC	SN	SP	PR
FS1					
ELM	Normal	90.93	90.39	92.18	90.84
	Mild	91.75	91.55	93.02	91.64
	Moderate	92.65	91.93	93.02	91.64
	Severe	93.73	92.84	94.17	93.66
	PDR	94.82	93.93	95.28	94.74
GWO-ELM	Normal	90.32	89.82	90.79	90.23
	Mild	91.57	91.06	92.53	91.40
	Moderate	92.40	92.60	92.97	92.44
	Severe	93.46	93.06	93.90	93.33
	PDR	94.54	93.82	94.73	94.44
BGWO-ELM	Normal	90.59	90.03	90.64	90.46
	Mild	90.66	90.35	91.84	90.56
	Moderate	91.93	91.34	92.69	92.10
	Severe	93.09	92.73	93.66	93.98
	PDR	94.17	93.69	94.62	94.08
KCBGWO_ELM	Normal	92.82	92.09	93.64	92.80
	Mild	93.72	92.04	94.63	93.69
	Moderate	94.65	93.93	95.40	94.74
	Severe	95.53	94.82	95.95	95.44
	PDR	96.48	95.74	96.90	96.35
**FS 2**					
ELM	Normal	91.74	91.45	92.82	92.36
	Mild	92.53	91.76	93.34	92.48
	Moderate	93.68	93.47	94.63	94.29
	Severe	94.75	94.08	95.84	95.14
	PDR	95.83	95.23	96.95	96.22
GWO-ELM	Normal	91.49	91.52	92.06	91.57
	Mild	92.67	91.75	93.46	92.75
	Moderate	93.84	93.33	94.70	93.95
	Severe	94.92	94.46	95.78	95.85
	PDR	96.00	95.55	96.82	96.78
BGWO-ELM	Normal	91.50	90.75	92.73	91.53
	Mild	92.76	92.17	94.09	92.90
	Moderate	93.97	93.28	94.63	93.83
	Severe	95.01	94.48	95.82	95.03
	PDR	96.19	95.64	96.80	96.08
KCBGWO_ELM	Normal	92.39	91.72	93.20	92.50
	Mild	93.62	93.06	94.73	93.79
	Moderate	94.83	94.27	95.62	95.00
	Severe	95.97	95.38	96.65	95.82
	PDR	97.10	96.53	97.73	97.09
**FS 3**					
ELM	Normal	92.76	92.10	93.43	92.66
	Mild	93.73	93.14	94.58	93.80
	Moderate	94.87	94.20	95.53	94.92
	Severe	95.93	94.46	96.28	95.85
	PDR	97.02	96.53	97.42	96.93
GWO-ELM	Normal	92.35	91.67	93.03	92.46
	Mild	93.59	92.93	94.35	93.66
	Moderate	94.74	94.08	95.40	94.85
	Severe	95.82	95.24	96.44	95.72
	PDR	96.89	96.32	97.50	96.78
BGWO-ELM	Normal	91.50	90.75	92.73	91.53
	Mild	92.76	92.17	94.09	92.90
	Moderate	93.97	93.28	94.63	92.83
	Severe	95.04	94.48	95.82	95.03
	PDR	96.18	95.64	96.80	96.08
KCBGWO_ELM	Normal	95.06	94.38	95.59	95.02
	Mild	96.28	95.59	96.73	96.35
	Moderate	97.45	96.73	97.90	97.43
	Severe	98.52	97.93	98.68	98.42
	PDR	99.15	98.64	98.28	99.01
**FS 4**					
ELM	Normal	93.88	93.28	94.53	93.98
	Mild	95.03	94.39	95.80	95.16
	Moderate	96.26	95.63	96.84	96.39
	Severe	97.32	96.73	97.95	97.20
	PDR	98.44	97.93	98.62	98.33
GWO-ELM	Normal	93.53	92.89	94.36	93.60
	Mild	94.74	94.20	95.53	94.88
	Moderate	95.97	95.39	96.65	96.1
	Severe	97.02	96.43	97.50	96.92
	PDR	98.17	97.64	98.73	98.08
BGWO-ELM	Normal	92.39	91.72	93.20	92.50
	Mild	93.62	93.06	94.73	93.79
	Moderate	94.83	94.27	95.62	95.00
	Severe	95.97	95.38	96.65	95.82
	PDR	97.10	96.53	97.73	97.09
KCBGWO_ELM	Normal	96.03	95.37	96.65	96.18
	Mild	97.22	96.64	97.76	97.36
	Moderate	97.62	97.36	97.77	97.56
	Severe	98.50	97.92	98.82	98.37
	PDR	99.04	98.56	99.28	99.23
**FuNet**					
ELM	Normal	95.33	94.46	94.75	95.00
	Mild	95.82	95.56	96.09	95.6
	Moderate	96.74	96.03	96.90	96.69
	Severe	97.63	97.13	98.09	97.55
	PDR	98.54	98.03	98.82	98.42
GWO-ELM	Normal	95.16	94.53	95.77	95.68
	Mild	96.64	95.59	96.78	96.56
	Moderate	97.50	96.49	97.63	97.43
	Severe	98.37	97.80	98.62	98.25
	PDR	99.03	98.47	98.97	98.88
BGWO-ELM	Normal	95.90	94.86	96.03	95.77
	Mild	96.88	96.10	97.23	96.86
	Moderate	97.74	96.86	97.89	97.69
	Severe	98.49	97.92	98.84	98.37
	PDR	99.04	98.56	99.32	98.93
KCBGWO_ELM	Normal	96.25	95.73	96.40	96.20
	Mild	96.95	96.25	97.09	96.86
	Moderate	98.52	97.94	98.76	98.20
	Severe	99.05	98.49	99.12	89.92
	PDR	99.87	99.33	99.78	99.43

The ELM classifier also proved to give good results in all the stages of diabetic retinopathy (DR). For normal cases, it has an accuracy (ACC) of 90.93%, and for other cases, such as mild, moderate, and severe stages, the accuracy remains impressive with values like 91.75%, 92.65%, and 93.73%, respectively. For PDR cases, the ELM classifier provided an accuracy of 94.82%, a sensitivity (SEN) of 93.93%, a specificity (SPC) of 95.28%, and a precision (PRE) of 94.74%.In the GWO-ELM classifier, the obtained ACC was 90.32% for normal cases, showing good performance. For PDR, the accuracy was slightly higher at 94.54%. This was also evident in the high SEN, SPC, and PRE values, indicating the success of this approach. The GWO-ELM maintained high metrics across all categories with values like 91.57%, 92.40%, and 93.46% for mild, moderate, and severe cases, respectively. The proposed BGWO-ELM classifier retained a high accuracy of at least 90.59% for normal cases and reached 94.17% for PDR cases. It demonstrated that the SEN, SPC, and PRE values remained high across all the stages, signifying that the classifier was reliable, with SEN ranging from 90.03% to 93.69%, SPC from 90.64% to 94.62%, and PRE from 90.46% to 94.08%. According to the experimental results, the proposed KCBGWO-ELM classifier had the highest accuracy for normal cases at 92.82% and for PDR cases at 96.48%. The analysis of the SEN, SPC, and PRE of KCBGWO-ELM also revealed that they were higher, indicating enhanced detection capability. The SEN reached as high as 95.74%, SPC at 96.90%, and PRE at 96.35% for PDR cases, showcasing its effectiveness. The FuNet classifier was found to have the highest overall accuracy compared to other models implemented. For normal cases, FuNet attained an average accuracy of 95.33%, a sensitivity of 94.46%, and a specificity of 94.75%, while the precision was 95.00%. In PDR cases, FuNet achieved an ACC of 98.54%, an SEN of 98.03%, an SPC of 98.82%, and a PRE of 98.42%. Such remarkable performances indicate that the proposed KCBGWO-ELM and FuNet classifiers could accurately detect DR at all the mentioned stages, demonstrating a promising strategy for clinical diagnosis and efficient management of related patients.

**Fig 12:**
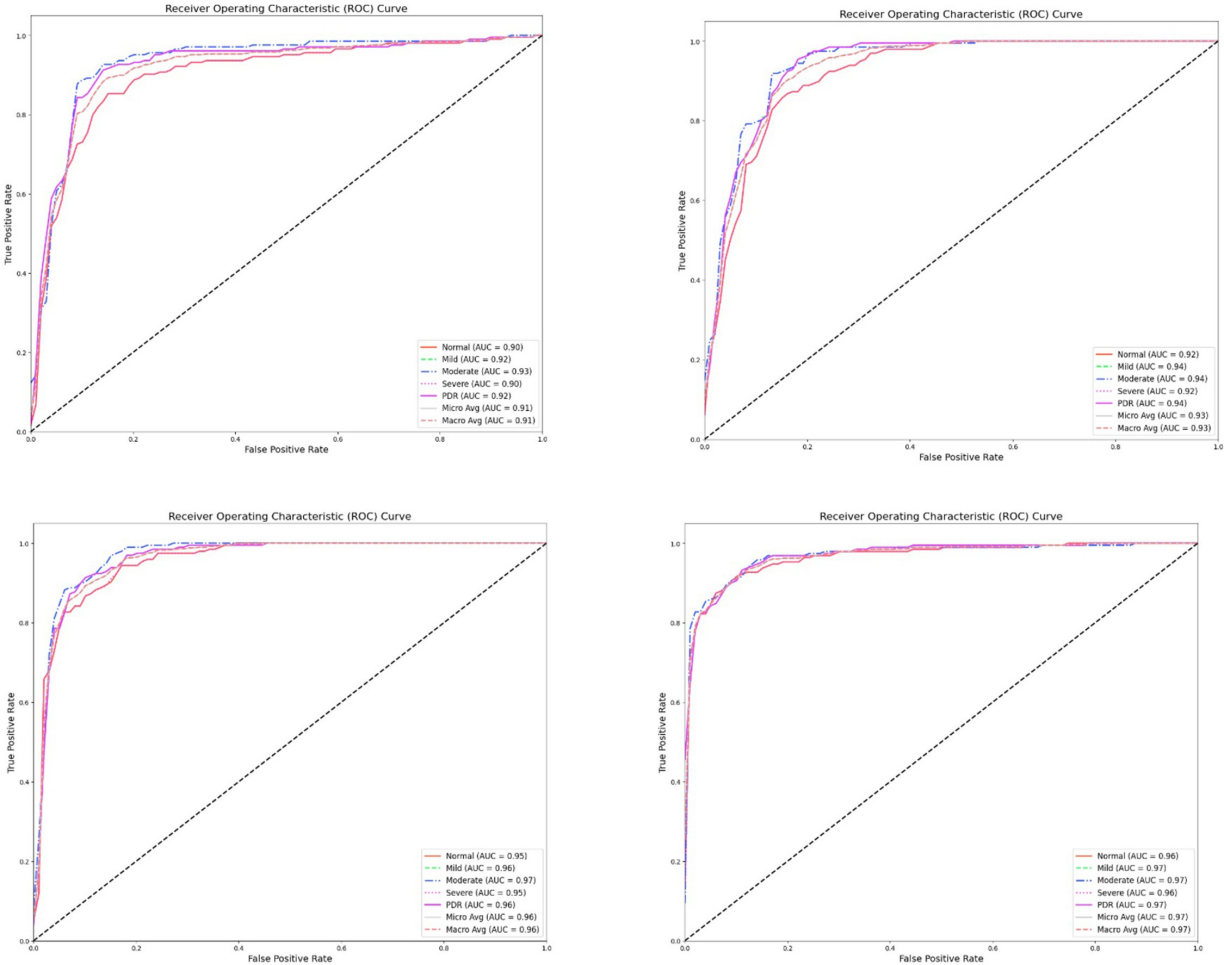
AUC for each stage.

The four ROC plots that were produced provide a thorough evaluation of the classifiers’ efficiency using four state-of-the-art approaches. KCBGWO-ELM, ELM, GWO-ELM, and BGWO-ELM are five separate classes that have been proposed: Normal, Mild, Moderate, Severe, and Proliferative Diabetic Retinopathy (PDR) are the levels of RD severity as shown below in Fig 12. Every method was tested on synthetic data generated with different random seeds to cover all aspects of each approach. The first plot for the ELM method showed that all the classifiers performed well. From the results in Fig 12, the Moderate class received the highest AUC of 0. 93, which is highly desirable for diagnostic tests because the instrument has a high discriminative ability. The model’s macro and micro average AUCs, which indicate equal classifier accuracy across all classes, were 0.91. The AUC values of 0 showed that the ROC curves for the other classes similarly had great levels of accuracy. Severe is 90, Normal is 90, and Mild is 0. 92 for PDR and Mild ethnicity. The second plot demonstrated that the suggested technique, GWO-ELM, much improved the classifier. An AUC of 0. 94 was also generated by the classifiers for the Mild, Moderate, and PDR classifications, demonstrating the improved accuracy and dependability of the approach. It is evident from comparing the provided ROC curves that the Normal and Severe classifications likewise had positive outcomes, with an AUC of 0.92. The recommended strategy was more consistent and trustworthy across all categories, as seen by the equal micro and macro average AUC of 0.93. The efficacy of the classifiers was displayed in the third plot of the suggested BGWO-ELM approach, with the moderate class having the highest 0.97AUC. The AUC for the Normal and Severe courses was 0.95, while the AUC for the Mild and PDR classes was 0.95. The micro and macro average AUCs, which are equal to 0.96, stated that the BGWO-ELM offered high performance without compromising between various classes, proving the method’s reliability. Last but not least, the 4^th^ plot of the proposed KCBGWO-ELM was quite promising, where Mild secures 0.97 AUC. Both Normal and Severe classes turned out to have an AUC of 0. 96. The AUC for micro and the macro average were both at 0. 97, meaning that the proposed method has been continuously and accurately classifying the given data.

Table 5 gives a comprehensive analysis of some of the techniques adopted in various categorization tasks, focusing on their performance metrics of precision, recall and F1-measure. It may be noted that in the context of the dynamic field of medical image analysis, FuNEt-based models like ELM, GWO-ELM, BGWO-ELM, and KCBGWO-ELM have offered a tremendous boost to diagnostic efficiency, especially in the case of IDRiD dataset. It would now be precise to say that the proposed model, KCBGWO-ELM, yields the best 99. 8% accuracy, 99.4% sensitivity and 99.9% Specificity. These metrics provide high diagnostic accuracy and an extraordinary ability to classify clinical and health cases.

**Table 5:** IDRiD-based state-of-the-art comparison.

Author	Method	Year	AC	SN	SP
[91]	Handcrafted Features +CNN	2019	90.7	.	.
[92]	GNN	2019	78.3	.	.
[93]	R-CNN	2019	.	83.0	94.0
[94]	CANet	2020	92.6	.	.
[95]	CNN	2020	90.3	88.8	96.9
		2020	90.9	88.8	96.3
[96]	RSNet	2020	86.3	.	.
[97]	CNN	2020	81.0	.	.
[7]	SVM	2021	98.1	83.7	100
[12]	Weighted CNN+GWO	2021	96.9	98.8	95.8
[98]	TL	2022	71.0	.	71.0
[99]	DCNN	2022	73.0	.	.
[100]	ELM	2022	99.0	.	.
[33]	CNN + SVD +Inception-V3	2022	97.9	-	-
[34]	GWO-CNN	2022	95.9	96.3	93.4
[101]	GNN	2023	96.0	.	.
[102]	CNN+UNet	2023	96.6	89.0	99.0
[103]	FTL+CNN	2023	92.2	90.1	85.8
[104]	Supervised contrastive learning	2023	98.9	.	.
[105]	MCNN	2023	90.1	.	93.8
[106]		2023	97.7	.	86.5
[107]	AHO-MLCNN	2023	97.8	.	.
[108]	Shallow CNN	2023	91.4	.	.
Proposed	ELM	2024	98.9	98.4	99.2
	GWO-ELM	2024	99.1	98.6	99.4
	BGWO-ELM	2024	99.2	98.7	99.5
	KCBGWO-ELM	2024	99.87	99.33	99.78

It is crucial to place this proposed model’s performance in perspective; for this reason, we will compare this model to other remarkable techniques until 2023. For example, a popular SVM model used in 2021 to analyse the IDRiD dataset provided high accuracy, equal to 98%. 06%, specificity of 77% and overall accuracy of 82%, while the second part yielded a sensitivity of 83%, specificity of 77%, and accuracy of 82%. The sensitivity was determined to be 67%, while the specificity of the test was determined to be 100%. Even though the SVM model has a specific value of 1, the sensitivity is much lower than that of the KCBGWO-ELM.Moreover, the accuracy was lower than the CNN+UNet model, developed in 2023, reaching 96. 50% with a specificity of 97 and sensitivity of 89. It also has a sensitivity of 100% and a specificity of 99%. In all aspects, the KCBGWO-ELM model has proved to have better results than the other models.

Other derivates of the proposed model, like the GWO-ELM and the BGWO-ELM, also yield impressive results. The GWO-ELM variant got an accuracy of 0.99. 12%, sensitivity of 98. 95% for the detection of breast cancer, with a sensitivity of 74% and specificity of 99%. 39%, while many of the preceding models are lower. Similarly, as in the case of the previous algorithm, its variant, namely the BGWO-ELM, was characterized by an accuracy of 99. 23%, sensitivity of 98. 75%, with a sensitivity of 100% and a specificity of 99. 51%, which can be seen as a better result than the performance indicators of other networks, such as 2023’s GNN and 2019’s R-CNN, which showed worse results in such measures.

Compared to the more modern methods, such as the supervised contrastive learning of the image data in 2023, which this paper was based on, it got an accuracy of 98. Overall, the accuracy of the proposed KCBGWO-ELM reaches 91%, and once again, the effectiveness of the developed algorithm in medical image diagnosis is of high level. This level of analysis establishes the FuNEt-based models as the innovation that defined the future of the field and created avenues for higher accuracy, sensitivity, and specificity, paving the way for the use of the models in actual clinical practice.

## Conclusion

This study proposes a novel approach for screening and classifying diabetic retinopathy utilizing modern neural network systems. Applying our proposed approach of combining Fully Convolutional Encoder-Decoder Networks (FCEDN) with K-Means Clustering- Binary Grey Wolf Optimizer (KCBGWO), the performance and accuracy of retinal image segmentation as well as analysis is greatly improved. Generative Adversarial Network (GANs) for synthetic data generation and transfer learning for feature extraction enhances the approach, providing unmatched reliability and robustness. Experimental results on the IDRiD dataset confirm the effectiveness of our proposed KCBGWO-ELM model for obtaining high accuracy, sensitivity, and specificity in DR diagnoses. These results reaffirm the efficacy and applicability of this method in reaching high accuracy in DR diagnosis early enough and in scalable methodologies, thus guaranteeing the patient better results. However, despite these promising results, our work has certain limitations. The dependency on high-quality annotated datasets like IDRiD restricts the generalizability of our model to other datasets and real-world scenarios with less comprehensive annotations. Additionally, the computational complexity of our approach may limit its deployment in resource-constrained environments.

Future work will address these limitations by incorporating more diverse and significant datasets to enhance the model’s robustness and generalizability. We also plan to explore the application of our framework to other ophthalmic conditions, leveraging advanced optimization techniques to boost performance further. Moreover, efforts will be directed towards optimizing the computational efficiency of our model, making it more accessible for broader clinical use, including in low-resource settings. This will ensure that our innovative approach can truly revolutionize DR detection and contribute to more effective and timely treatment strategies.
